# A WRKY transcription factor PbrWRKY53 from *Pyrus betulaefolia* is involved in drought tolerance and AsA accumulation

**DOI:** 10.1111/pbi.13099

**Published:** 2019-03-19

**Authors:** Yue Liu, Tianyuan Yang, Zekun Lin, Bingjie Gu, Caihua Xing, Liangyi Zhao, Huizhen Dong, Junzhi Gao, Zhihua Xie, Shaoling Zhang, Xiaosan Huang

**Affiliations:** ^1^ College of Horticulture State Key Laboratory of Crop Genetics and Germplasm Enhancement Nanjing Agricultural University Nanjing China; ^2^ State Key Laboratory of Tea Plant Biology and Utilization Anhui Agricultural University Hefei China

**Keywords:** *Pyrus betulaefolia*, abiotic stress, ascorbic acid, antioxidant, transcriptional regulation

## Abstract

WRKY comprises a large family of transcription factors in plants, but most WRKY members are still poorly understood. In this study, we report the identification and functional characterization of *PbrWRKY53* isolated from *Pyrus betulaefolia*. *PbrWRKY53* was greatly up‐regulated by drought and abscisic acid, but slightly induced by salt and cold. Subcellar localization analyses showed that PbrWRKY53 was located in the nucleus. Ectopic expression of *PbrWRKY53* in tobacco and *Pyrus ussuriensis* conferred enhanced tolerance to drought stress. The transgenic plants exhibited better water status, less reactive oxygen species generation and higher levels of antioxidant enzyme activities and metabolites than the wild type. In addition, overexpression of *PbrWRKY53* in transgenic tobacco resulted in enhanced expression level of *PbrNCED1*, and led to the increase in larger amount of vitamin C accumulation in comparison to WT. Knock‐down of *PbrWRKY53* in *P. ussuriensis* down‐regulated *PbrNCED1* abundance, accompanied by compromised drought tolerance. Yeast one‐hybrid assay, EMSA and transient expression analysis demonstrated that PbrWRKY53 could bind to the W‐box element in the promoter region of *PbrNCED1*. Taken together, these results demonstrated that *PbrWRKY53* plays a positive role in drought tolerance, which might be, at least in part, promoting production of vitamin C via regulating *PbrNCED1* expression.

## Introduction

Drought stress is one of the most major environmental limited factors that seriously hampers crop productivity. Therefore, it is urgent to enhance drought tolerance for increasing agricultural productivity to meet the food demand of expanding population. Genetic engineering by expressing important genes involved in drought‐responsive, which has been shown to act as an effective approach for developing transgenic plants with enhanced drought tolerance (Zhang *et al*., 2018; Zhu, 2016). In this regard, it is a critical need for understanding the physiology and molecular mechanism underlying of plants to cope with drought stress.

It has been documented that plants respond and adapt to drought stress through modifying transcriptional levels of a large number of genes (Seki *et al*., 2001, 2002). Due to the different product of these genes can be divided into two categories, effector molecules or regulator molecules. The effector molecule produced by drought resistant genes can keep the protective enzyme activity at a high level under adverse conditions, which is beneficial to scavenge reactive oxygen species (ROS), alleviate cell damage. The other mechanism is to regulate the expression of related genes by regulating protein. Plants regulate a large spectrum of stress‐responsive genes involved in the synthesis of various metabolites that combat the abiotic stresses. Abscisic acid (ABA) is well known as a multi‐function phytohormone which is critical for diverse physiological and developmental processes and plays an important role in the plant response to various environmental stresses (Fujita *et al*., 2011). As an adversity signal, ABA plays an important role in many plant abiotic stresses such as drought, salinity, chilling, especially in drought tolerance, which is mainly due to the induction in the genes responsible for ABA synthesis (Hu and Xiong, 2014; Tardieu *et al*., 2018; Xiong and Zhu, 2003). Drought leads to stomatal closure to prevent water loss through reducing transpiration. Since the content of ABA is closely related to degree of stomatal opening and closing, plants can strictly regulate the ABA concentration when are posed to stress conditions such as drought, chilling and salt (Hu and Xiong, 2014). 9‐*cis*‐ type carotenoid bioxygenase (NCED) is the key enzyme in the synthesis of ABA under drought stress, and the over‐expression of *NCED* gene can enhance the ability of drought resistance in plants with ABA accumulation (Xian *et al*., 2014), while *AtNCED3* knockout lines showed drought‐sensitive phenotype with decreased ABA level (Iuchi *et al*., 2001). An earlier report demonstrated that co‐expression *SgNCED1* from a forage legume and *ALO* from yeast in tobacco and stylo could increase vitamin C level and improve tolerance of drought and chilling (Bao *et al*., 2016).

Plant cells possess a complex antioxidant defense system for alleviating various abiotic stresses under unfavourable environment, which consists of non‐enzymatic antioxidants and ROS‐scavenging enzymes (Miller *et al*., 2010). The functions of these two antioxidant defense systems are scavenging the elevated ROS, including hydrogen peroxide (H_2_O_2_) and hydroxyl radicals, $O_{2}^{-}$ (superoxide radicals), in plant cells caused by abiotic stresses, which is responsible for protection against oxidative damages on cell membranes. Of these, Superoxide dismutase (SOD) provides the first line of defense against ROS by catalyzing the dismutation of $O_{2}^{-}$ to oxygen and H_2_O_2_, which was then scavenged by coordinated action of CAT, POD, APX, ascorbic acid (AsA) and glutathione (GSH) (Blokhina *et al*., 2003; Gill and Tuteja, 2010). AsA, as a cofactor for many enzymes, contributes to plant growth, cell division development and abiotic stress responses by scavenging ROS produced as a by‐product of photorespiration (Chen and Gallie, 2005; Conklin, 2001). AsA is synthesized through multiple biosynthetic pathways in plants. Among them, AsA‐GSH is the major pathway including DHAR1, MDHAR and APX, can scavenge H_2_O_2_ by converting it into H_2_O by AsA. The DHAR protein catalyzes DHA to AsA in AsA‐GSH cycle. Previous report indicated that overexpression *SgNCED1* significantly improved DHAR activities and AsA contents in tobacco faced with high concentration ozone (Chen and Gallie, 2005). Although it has been reported the *NCED1* may contribute to regulating AsA levels, the molecular regulatory mechanism of gene transcript level is still poorly understood.

Transcription factors (TFs) act as significant coordinators to transducer stress signals and to orchestrate expression of their target genes. It plays crucial roles in providing protection against stress‐associated damage by modulating expression level of downstream target genes (Vigeolas *et al*., 2008). Extensive evidences have been demonstrated overexpression of TF gene may activate a group of target genes that function in a concerted manner to counteract the adverse effects of abiotic stresses (Schluttenhofer and Yuan, 2015). Therefore, genetic engineering of TFs has been proposed to be a robust strategy for improving the stress tolerance of crop plants (Golldack *et al*., 2011; Huang *et al*., 2013). More than 1500 TFs accounting for nearly 6% of its total genes have been identified in the genome of Arabidopsis (Gong *et al*., 2004). Among these TFs, the WRKY gene family is an important TF family of plants and plays an essential role in resistance of stress responsive signalling pathways. WRKY protein is a new type of plant specific zinc finger transcriptional regulator, which is named after its N‐terminal contains a highly conserved seven amino acid sequences composed of WRKYGQK (Eulgem *et al*., 2000). WRKY proteins are defined by presence of one or two conserved domains, called WRKY domain (Rushton *et al*., 2010). WRKY TF gene is induced by both biotic and abiotic stresses, and is involved in plant stress responsive. The WRKY TFs are a large gene family in the plant genome, there are 74 genes in *Arabidopsis*, more than 100 in rice, 119 in corn, 104 in *Populus tremula* and 46 in rapeseed (Eulgem *et al*., 2000; He *et al*., 2012; Yang *et al*., 2009; Zhang and Wang, 2005). WRKYs have been considered as critical TFs involved in biotic stress response, while emerging experimental data provide evidence to support the implication of WRKYs in the regulation of abiotic stress responses (He *et al*., 2016; Niu *et al*., 2012; Zhou *et al*., 2008). Furthermore, some WRKY TFs are especially responsive to drought stress in plant. For example, *FcWRKY70*, a *Fortunella crassifolia* WRKY gene, functions in drought tolerance and modulates putrescine synthesis by regulating arginine decarboxylase gene (Gong *et al*., 2015). The *BdWRKY36* from *Brachypodium distachyon* confered tolerance to drought stress in transgenic tobacco plants (Sun *et al*., 2015). Recently, another two WRKY genes, *ABO3*,* ThWRKY4*, have also been shown to be key regulators of drought responses by modulation of drought responsive genes under drought stress (Ren *et al*., 2010; Zheng *et al*., 2013). Overexpression of *GsWRKY20* from soybean improved drought stress tolerance through decreasing the stomatal density and enhanced stomatal closure in transgenic soybean (Ning *et al*., 2017). Epitopic expression of *MuWRKY3* dramatically enhanced the drought tolerence of *Arachis hypogaea* via accumlating less malondialdehyde, hydrogen peroxide (H_2_O_2_) and superoxide anion accompanied by increasing the content of proline, soluble sugar content and activities (Kiranmai *et al*., 2018).

Although physiological functions of some WRKY TFs have been characterized in model plants, the functions of most of WRKY TFs are still poorly understood, especially in non‐model plants, such as *Pyrus betulaefolia*. In this study, we report the identification and functional characterization of a novel TF gene, *PbrWRKY53*, which directly binds to the *PbrNCED1* promoter. We also demonstrated that PbrWRKY53 is a positive regulator of *PbrNCED1* expression and AsA biosynthesis. Furthermore, the present results in our study suggested that PbrWRKY53 acts as a positive regulator of drought tolerance, which was at least in part through modulation of AsA‐mediated ROS scavenging.

## Results

### Isolation and bioinformatics analysis of *PbrWRKY53*

**Figure 1:**
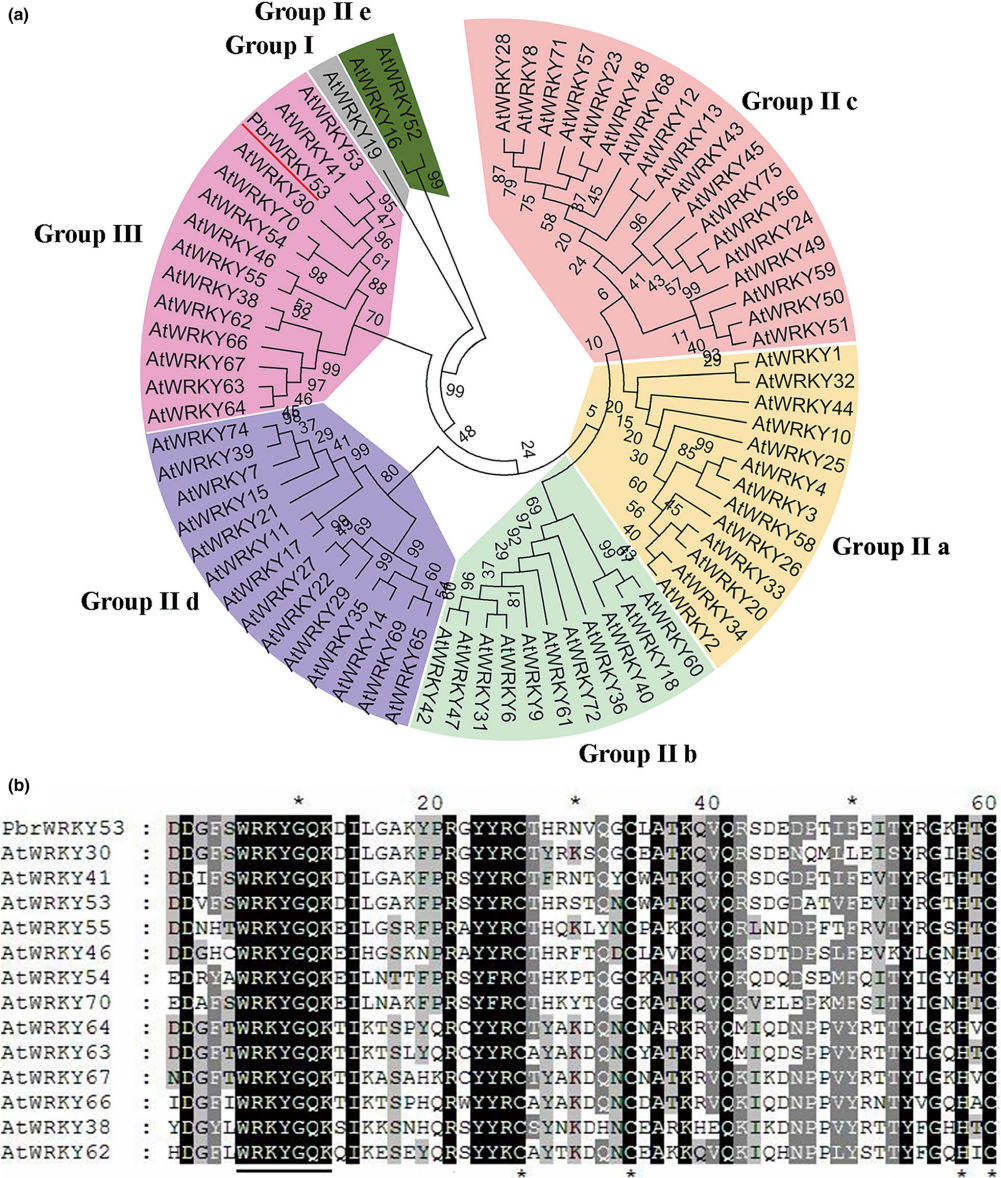
Phylogenetic analysis and multiple alignments of WRKYs from different plants. (a) A phylogenetic tree constructed based on WRKYs of Arabidopsis thaliana and PbrWRKY53. (b) Multiple alignments of the conserved WRKY domains between the Group III WRKYs. Identical amino acids are shown with a black background and analogous amino acids are shaded in gray. The WRKY motif is shown by a line while the zinc‐finger structures are represented by asterisks.

We previously obtained a remarkably drought‐induced WRKY TF (Pbr018725.1) named *PbrWRKY53* from a transcriptome of *P. betulaefolia* (Li *et al*., 2016). Sequencing results indicate the full length of cDNA is 1746 bp in size. Bioinformatics analysis show that PbrWRKY53 contains a 1041 bp open reading frame (ORF) and encodes a predicted polypeptide of 347 amino acids with a calculated molecular mass of 38.7 kDa and an isoelectric point of 5.8. The full‐length sequence of PbrWRKY53 shared high homology with MdWRKY53 (80%); the gene was thus designated as PbrWRKY53. A phylogenetic tree constructed based on the PbrWRKY53 and using a total of 72 WRKYs from *Arabidopsis* shows that they could be classified into four major groups, and PbrWRKY53 belongs to the Group III (Figure 1a). Multiple sequence alignment suggested that PbrWRKY53 protein has a highly conserved WRKY domain composed of 60 amino acids (140–199) (Figure 1b).

### Expression patterns of *PbrWRKY53* in response to various abiotic stresses

**Figure 2:**
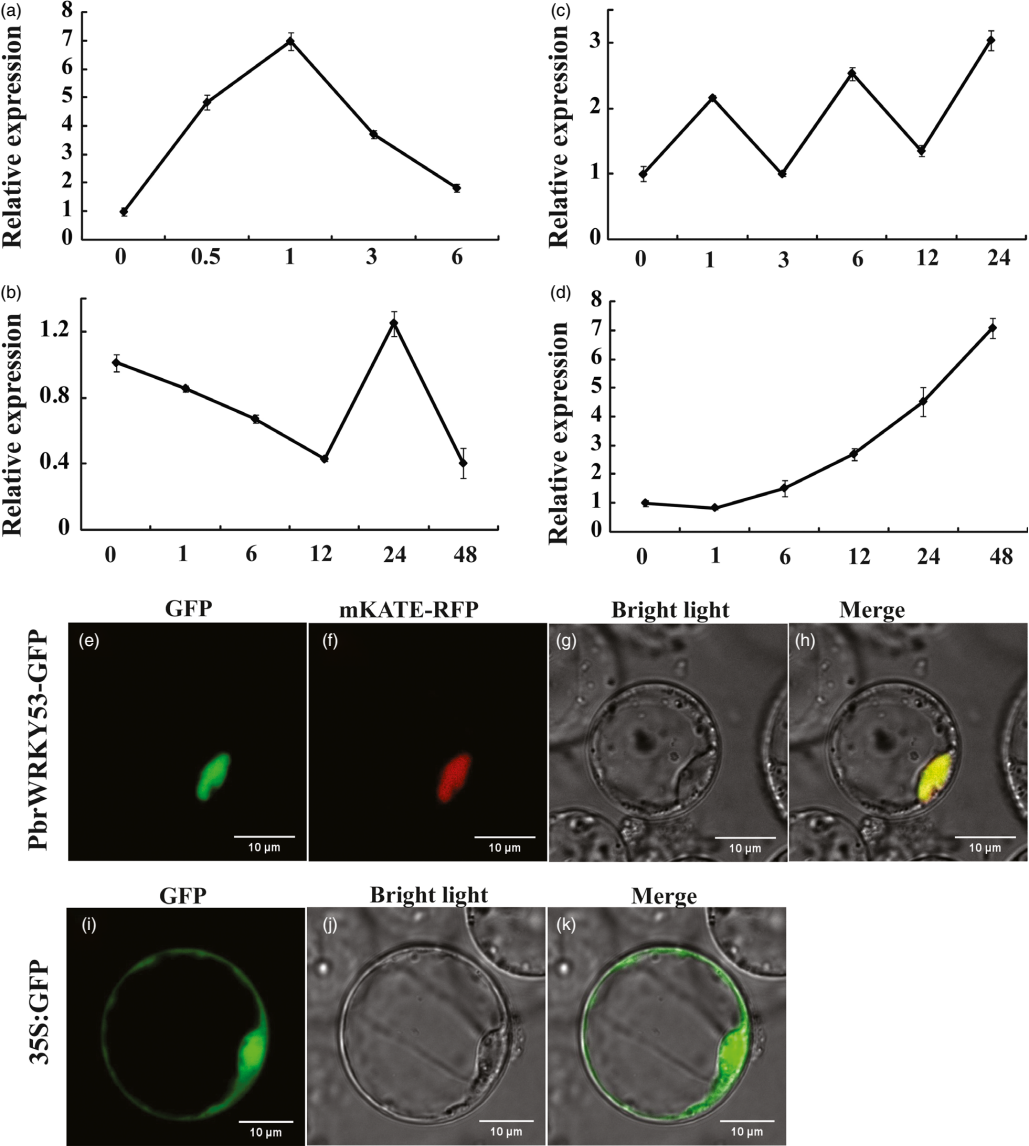
Analysis of expression patterns and subcellular localization of PbrWRKY53.Time‐course expression levels of *PbrWRKY53* under different treatments with dehydration (a), low temperature (b), salt (c) and ABA (d). For each treatment, the expression level at 0 h was set as 1.0 and data represented means ± SE of three replicates. Subcellular localization of PbrWRKY53. Rice protoplasts cells were transiently transformed with constructs containing fusion plasmid (PbrWRKY53: GFP, e). Images under bright field and merge were shown in f, g, respectively. Rice protoplasts cells were transiently transformed with construct containing vector plasmid (GFP, i). Images under bright field and merge were shown in j, k, respectively.

The expression patterns of *PbrWRKY53* under different abiotic stresses, including dehydration, cold, salt and ABA, was analysed by qPCR. As shown in Figure 2a, *PbrWRKY53* transcript level quickly accumulated at 0.5 h after dehydration, and steady continued to induce, reaching a maximum at 1 h (greater than sevenfold induction), while exhibited a slight decrease at the last time point. The transcript abundance of *PbrWRKY53* was down‐regulated under cold treatment for 1 h, and decrease quickly to the low value at 12 h (Figure 2b). The result indicated that PbrWRKY53 was repressed by cold stress at early stage. Earlier reports revealed that long periods of low temperature could also cause cell dehydration (Ding *et al*., 2019). Our result showed that *PbrWRKY53* was drought‐inducible. We suppose that the expression level of *PbrWRKY53* increased by more than 1.4 folds at 24 h to response to the dehydration signal with the prolonging of cold stress time (Figure 2b). After long‐term cold treatment, the mRNA degraded gradually in plants, therefore the expression of *PbrWRKY53* declined eventually at 48 h of cold stress. When subjected to salt treatment, the transcript of *PbrWRKY53* did not noticeably alter the expression level of the gene (Figure 2c). In the case of ABA treatment, the transcript level of *PbrWRKY53* was slightly down‐regulated at 1 h time point, then it was induced dramatically more than sevenfold of the initial level until the end of treatment (Figure 2d). The dehydration treatment caused a greater induction compared with cold, salt and ABA, indicating that *PbrWRKY53* might perform important functions against the abiotic stresses, particularly drought.

### Subcellular localization of PbrWRKY53

Subcellular localization of PbrWRKY53 was investigated by examining a PbrWRKY53‐GFP fusion protein. The full‐length ORF of PbrWRKY53 was fused to the N‐terminal of GFP reporter protein of pCAMBIA1302 vector driven by CaMV 35S promoter, generating a fusion construct PbrWRKY53::GFP. The GFP‐PbrWRKY53 and red fluorescent protein (RFP)‐mKATE were cotransformed into rice protoplasts, we used a nuclear signal peptide fused to a RFP protein mKate (Shcherbo *et al*., 2007) as a positive control. Microscopic visualization demonstrated that the PbrWRKY53‐fusion protein fluorescence perfectly overlapped with RFP fluorescence (Figure 2e–h), indicating that PbrWRKY53 was localized in the nucleus. However, green fluorescence was exclusively detected in the entire cell region when the only GFP plasmid was transformed into rice protoplasts (Figure 2i–k).

### Overexpression of *PbrWRKY53* modulated stomatal aperture and alteration of endogenous ABA and AsA contents in transgenic plants

**Figure 3:**
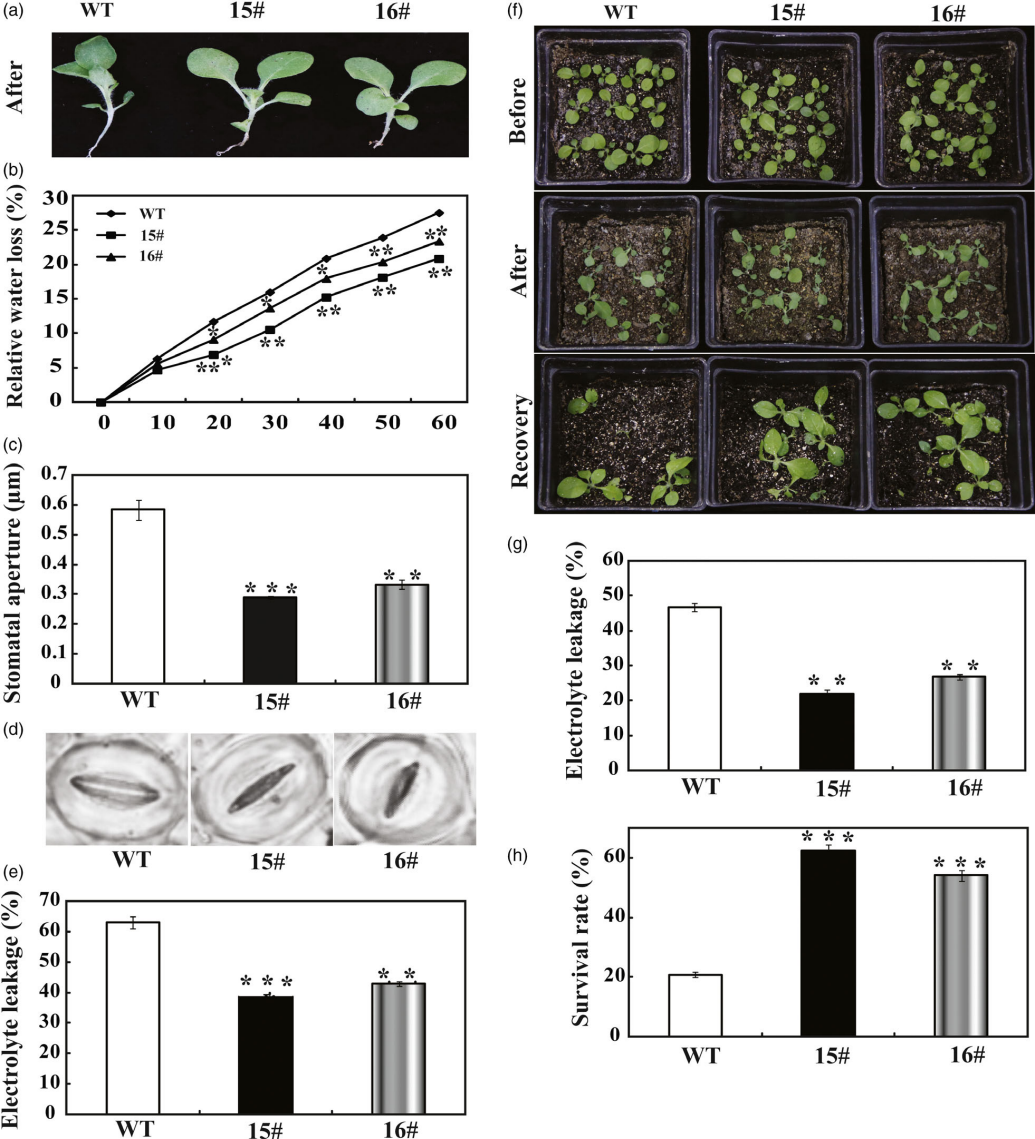
Determination of drought tolerance of *PbrWRKY53*‐overexpressing plants. (a) A representative photograph showing the dehydrated seedlings. (b) Time‐course fresh water loss of WT, transgenic lines (15# and 16#) during a 60‐min dehydration. (c and d) Stomatal aperture size (c) and representative images (d) of WT, 15# and 16# after dehydration treatment. (e) Electrolyte leakage (EL) of WT, 15# and 16# after dehydration for 60 min. (f) Plant phenotype of transgenic lines and WT before (upper panel) and after a 15 days water deprivation treatment (middle panel) and after 3 days recovery period (lower panel). (g) EL of WT, 15# and 16# after drought for 15 days. (h) Survival rates of transgenic lines and WT scored after the recovery. The data were analysed by Duncan's multiple range tests in the ANOVA program of SPSS (IBM SPSS 22). Asterisks show that the values are significantly different between the transgenic lines and the WT at the same time point (*P < 0.05; **P < 0.01; ***P < 0.001).

As *PbrWRKY53* transcript level was induced by dehydration in a stronger manner, transgenic tobacco plants overexpressing *PbrWRKY53* were generated to characterize the role of *PbrWRKY53* in either short‐term dehydration or long‐term drought tolerance. Two overexpression of *PbrWRKY53* lines (hereafter designated as 15# and 16#, Figure S1) with higher transcript levels of *PbrWRKY53* were used for the stress tolerance test. When the 30‐day‐old *in vitro* seedlings were dehydrated in an ambient environment, WT leaves displayed more serious wilting compared with the transgenic leaves (Figure 3a). The transgenic lines lost remarkably less water than the WT during any time point within 60 min of dehydration (Figure 3b). At the end of dehydration, stomatal apertures of transgenic lines were significantly smaller about 40%–46% than those of WT (Figure 3c,d). Electrolyte leakage (EL), is an important indicator of cell injury. In our study, the EL was only approximate 43%–53% in transgenic tobacco compared to WT (Figure 3e), suggesting that membrane damage of WT lines was more serious than transgenic lines of tobacco by expressing *PbrWRKY53*.

**Figure 4:**
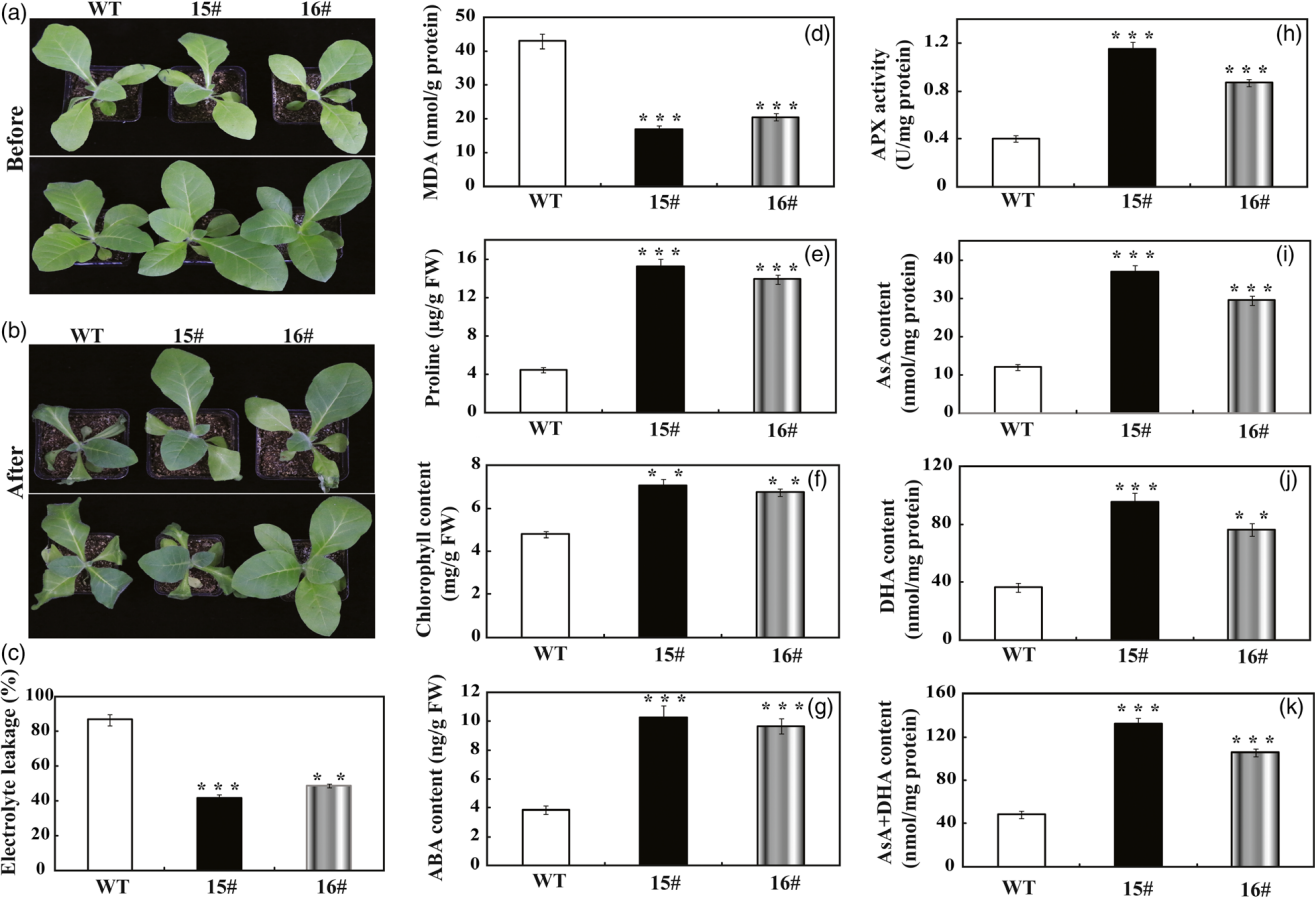
Phenotype and drought tolerance of wild type (WT) and transgenic lines (15# and 16#). (a and b) Representative photographs of potted plants of WT, 15# and 16# that have been exposed to water stress for 0 day (a) and 15 days (b). (c–f) Electrolyte leakage (c), MDA levels (d), proline contents (e) and total chlorophyll content (f) of WT, 15# and 16# after 15 days of water stress. (h–k) Analysis of APX content (h), ascorbic acid content (i), dehydroascorbate (DHA) content (j) and AsA + DHA content (k) of transgenic lines and WT after drought stress. The data were analysed by Duncan's multiple range tests in the ANOVA program of SPSS (IBM SPSS 22). ** and *** indicate that values of the two transgenic lines were significantly different from those of WT at *P* < 0.01 and *P* < 0.001, respectively.

To assess the effect of overexpression *PbrWRKY53* in tobacco on drought tolerance, the 18‐day‐old WT and transgenic plants were exposed to drought stress by withholding water for 15 days. After 15 days without water, the WT plants showed more sensitivity to the drought stress, as manifested by stronger leaf‐wilting symptoms, a higher EL and a lower survival rate, compared with the two transgenic lines (Figure 3f–h). In another experiment, 65‐day‐old plants were subjected to the treatment of drought stress by withholding for 7 days (Figure 4a). Similarly, morphological appearances of the transgenic plants were better than that of the WT, as manifested by presence of more dead leaves in the latter (Figure 4b). EL of 15# (42.0%) and 16# (48.8%) was significantly lower in comparison with 86.8% of WT (Figure 4c). In agreement with the enhanced drought susceptibility, the WT had greater values of malondialdehyde (MDA) following exposure to drought condition than did the transgenic plants (Figure 4d). Meanwhile, the Pro contents of the 15# and 16# were significantly higher than that of the WT (Figure 4e). Chlorophyll content of transgenic lines was significantly higher than that of WT (Figure 4f). It has been well documented that the content of ABA is closely related to drought stress of plants. Therefore, ABA levels of the WT and transgenic lines were quantitatively examined in this study. As shown in Figure 4g, ABA levels of the transgenic lines were 2.50–2.67 folds higher than that of WT under drought stress. Previous studies showed that stress‐induced ABA accumulation increased the levels of AsA (Chen *et al*., 2015; Zhang *et al*., 2009), which prompted us to examine the AsA contents in transgenic lines. In agreement with our expected, APX activity in transgenic plants was progressively higher than that of WT (Figure 4h). It should be noticeable that the AsA contents in the transgenic lines were 2.43–3.06 folds higher than that of WT, and the transgenic plants had higher levels of DHA, and AsA+DHA in comparison to WT plants (Figure 4i–k).

**Figure 5:**
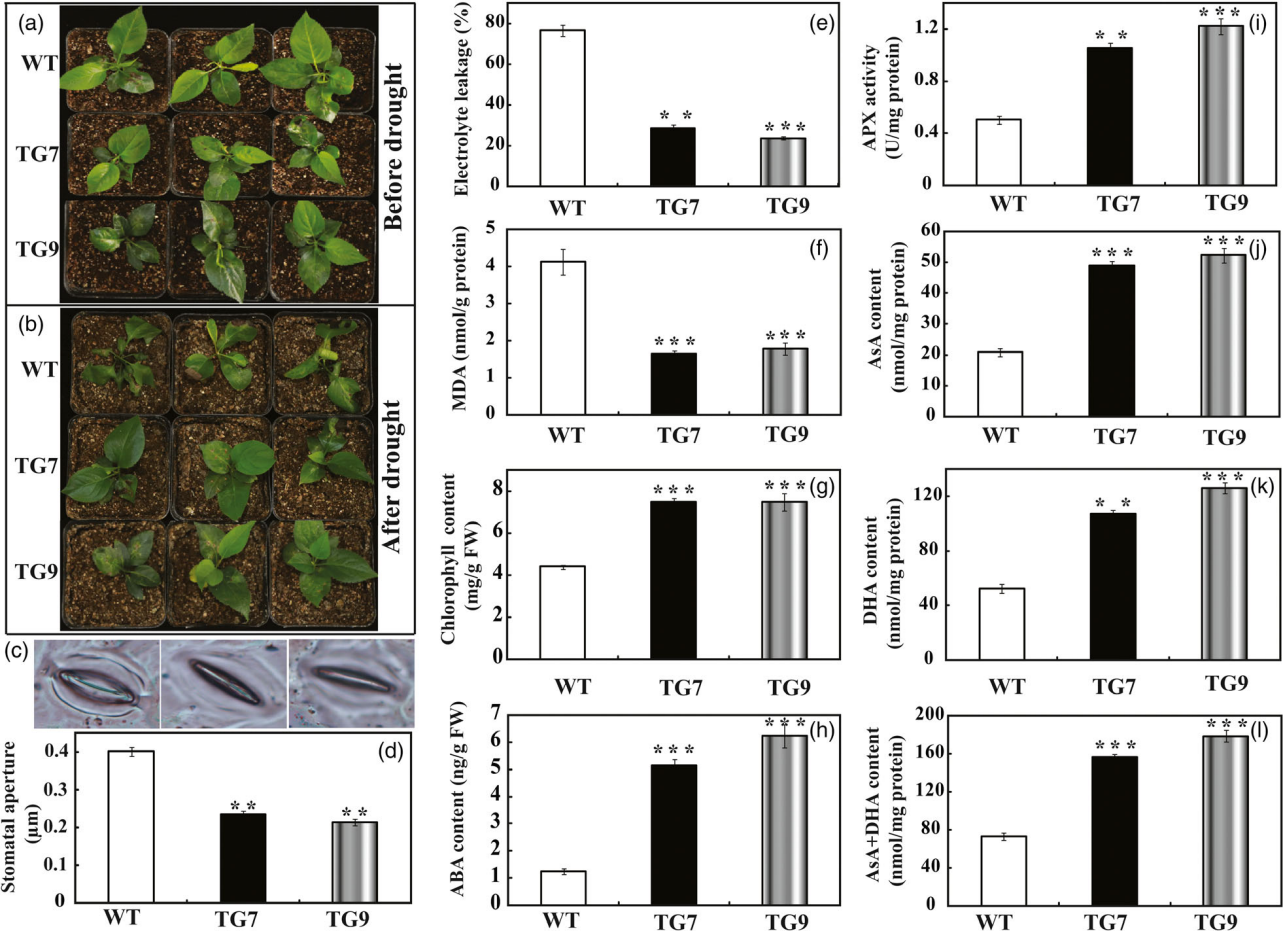
Drought tolerance assay of transgenic *Pyrus ussuriensis* plants. (a and b) Phenotypes of *P. ussuriensis* transgenic plants (TG7 and TG9) and the wild type (WT) before and after drought stress. (c and d) Stomatal representative images (c) and relative aperture size (d) of WT, TG7 and TG9 after drought treatment. (e‐h) Electrolyte leakage (e), MDA content (f), Chlorophyll content (g) and ABA content (h) of wild‐type *P. ussuriensis* and transgenic plants analyzed after drought treatment. (i–l) Analysis of APX content (i), ascorbic acid content (j), dehydroascorbate (DHA) content (k) and AsA + DHA content (l) of transgenic *P. ussuriensis* lines and WT after drought stress. The data were analysed by Duncan's multiple range tests in the ANOVA program of SPSS (IBM SPSS 22). ** and *** indicate that values of the two transgenic lines were significantly different from those of WT at *P* < 0.01 and *P* < 0.001, respectively.

*PbrWRKY53* was also transferred into *Pyrus ussuriensis* to further characterize its role in drought tolerance. Two transgenic *P. ussuriensis* lines (designated as TG7 and TG9) exhibiting a higher abundance of *PbrWRKY53* protein, were used for drought tolerance test (Figure S2). When the 35 days old wild‐type and transgenic *P. ussuriensis* plants were exposed to drought stress by withholding water for 17 days, the WT plants showed visual symptoms of leaf rolling, wilting and necrosis, whereas the transgenic lines appeared better growth (Figure 5a,b). The stomatal apertures of WT were significantly larger than those of the transgenic lines after the drought treatment (Figure 5c,d), which is consistent with the drought phenotype. In order to compare the physiological differences, we measured EL, MDA and total chlorophyll content, three widely used indicators of damage caused by abiotic stresses. EL in the transgenic lines (28.7% for TG7 and 23.7% for TG9) was significantly lower than the 76.6% of the wild type (Figure 5e). In addition, the transgenic lines had significantly lower MDA levels compared to the WT (Figure 5f), while higher total chlorophyll contents were observed in two transgenic lines (7.47 μg/g FW and 7.49 μg/g FW for TG7 and TG9, respectively) than in the wild type (4.41 μg/g FW for WT, Figure 5g). As ABA is an important phytohormone involved in drought tolerance (Xian *et al*., 2014), we attempted to check whether the ABA content was altered in the tested plants. The ABA contents in the transgenic lines were approximately sevenfold higher than that of WT (Figure 5h). In line with the results of transgenic plants in tobacco, the enzyme activity of APX in transgenic lines of *P. ussuriensis* was about 2.5–3 times higher than that of WT. Similarly, the AsA content, DHA content and AsA + DHA content were significantly higher than those of WT (Figure 5i–k). Taken together, these data demonstrated that overexpression of *PbrWRKY53* elevated endogenous both ABA and AsA levels in the transgenic plants and enhance drought tolerance of plants.

### Silencing of *PbrWRKY53* in *P. ussuriensis* confers sensitivity to drought stress

To further elucidate the role of *PbrWRKY53* in drought tolerance, we attempted to knock down the *PbrWRKY53* of *P. ussuriensis* using a virus‐induced gene silencing (VIGS). Transcript analysis of the leaflets revealed that the transcripts for *PbrWRKY53* were suppressed in the respective silenced plants (Figure S3a). Six VIGS plants in which *PbrWRKY53* was similarly suppressed were pooled to constitute a VIGS line, hereafter referred to as pTRV2‐PbrWRKY53 silencing plants (pTRV2‐1 and pTRV2‐2). When subjected to drought treatment for 17 days, the pTRV2‐PbrWRKY53 silencing plants displayed more serious wilting and compared with the WT (Figure S3b). At the end of drought, stomatal apertures of pTRV2‐PbrWRKY53 silencing plants were significantly larger than those of the WT (Figure S3c,d), consistent with phenotype. EL and MDA concentrations of the pTRV‐PbrWRKY53 plants were significantly higher than the WT plants at the end of drought (Figure S3e,f). Conversely, the AsA content in the two silencing plants (pTRV2‐1 and pTRV2‐2) were significantly lower than those of the WT after drought treatment. We then measured transcript level of the *PbrNCED1* in the pTRV2‐PbrWRKY53 silencing plants, and found that this gene was prominently down‐regulated relative to those of the WT (Figure S3h). These results indicated that silencing of *PbrWRKY53* by VIGS elevated drought sensitivity in *P. ussuriensis*.

### Transgenic lines accumulate less ROS and exhibit higher antioxidant enzyme activities under drought stress

**Figure 6:**
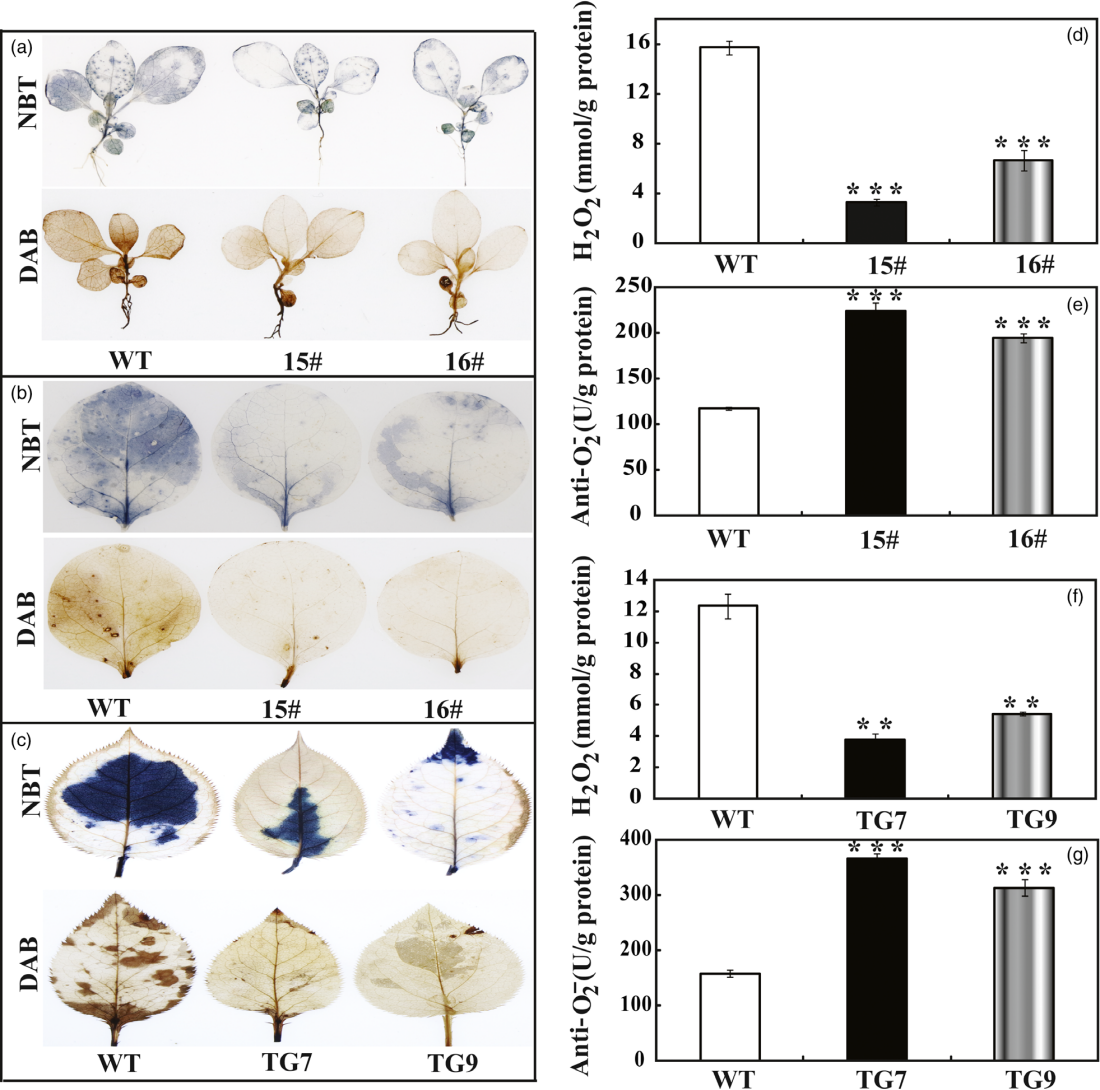
Analysis of H_2_O_2_ and anti‐$O_{2}^{-}$ in transgenic plants (tobacco and *Pyrus ussuriensis* after dehydration or drought stresses. (a) Histochemical staining with DAB and NBT for detection of H_2_O_2_ and $O_{2}^{-}$, respectively, in WT, 15# and 16# after 60 min of dehydration. (b) Representative photos showing accumulation of $O_{2}^{-}$ (upper panel) and H_2_O_2_ (lower panel) in tobacco WT and transgenic lines (15# and 16#) after 7 days water stress. (c) Representative images indicating *in situ* accumulation of $O_{2}^{-}$ (upper panel) and H_2_O_2_ (lower panel) in *Pyrus ussuriensis* WT and transgenic lines (TG7 and TG9) after drought stress for 7 days. (d and e) Levels of $O_{2}^{-}$ (d) and H_2_O_2_ (e) in tobacco WT and transgenic lines (15# and 16#) after drought stress. (f–g) Levels of $O_{2}^{-}$ (f) and H_2_O_2_ (g) in *P. ussuriensis* WT and transgenic lines (TG7 and TG9) after drought treatment. The data were analysed by Duncan's multiple range tests in the ANOVA program of SPSS (IBM SPSS 22). ** and *** indicate that values of the two transgenic lines were significantly different from those of WT at *P* < 0.01 and *P* < 0.001, respectively.

**Figure 7:**
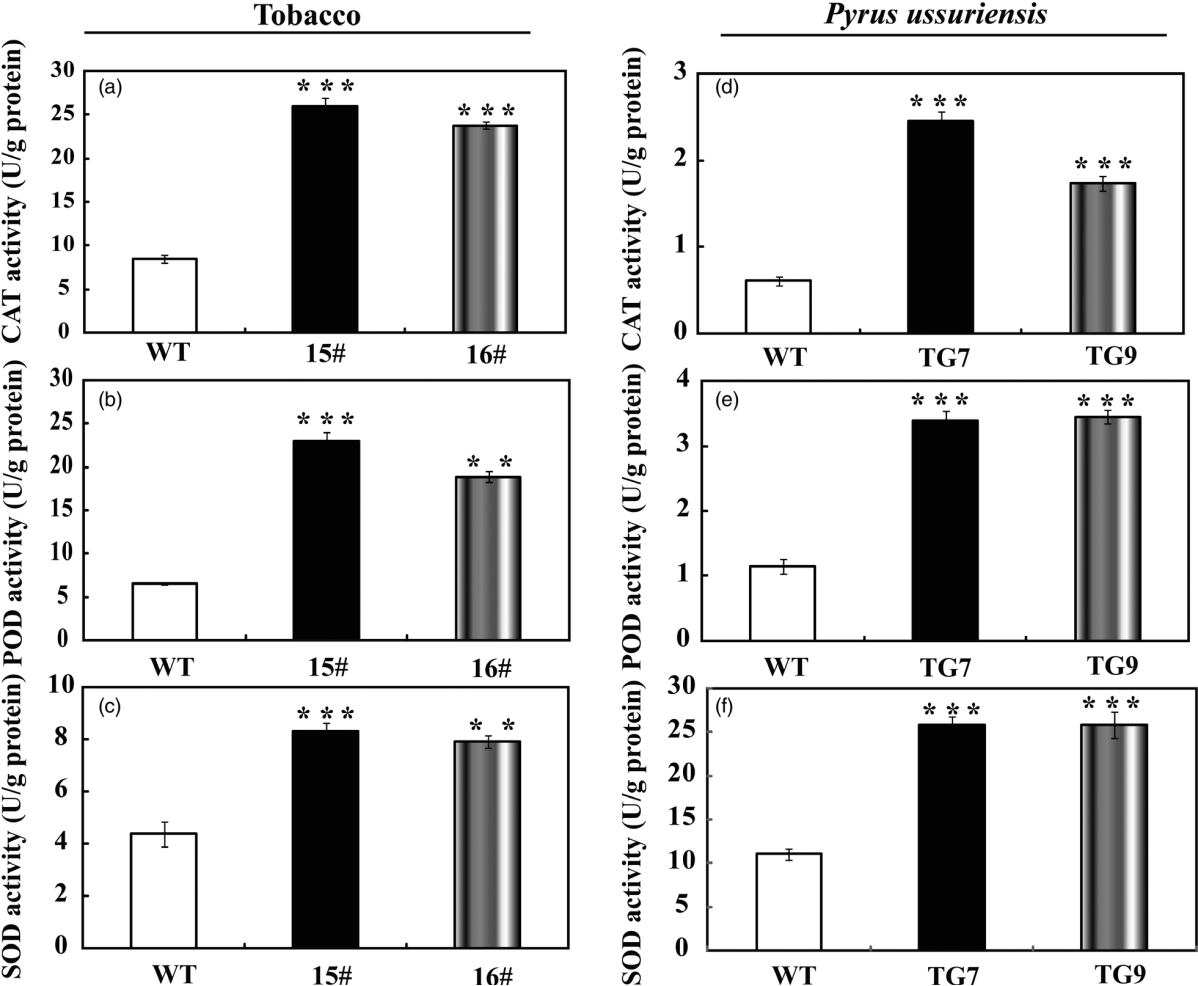
Analysis of antioxidant enzymes activities in tobacco and *Pyrus ussuriensis*. (a–c) Activity of CAT (a), POD (b) and SOD (c) in tobacco WT and transgenic lines (15# and 16#) before and after drought treatment. (d–f) Activity of CAT (d), POD (e) and CAT (f) in *P. ussuriensis* WT and transgenic lines (TG7 and TG9) before and after drought stress. The data were analysed by Duncan's multiple range tests in the ANOVA program of SPSS (IBM SPSS 22). ** and *** indicate that values of the two transgenic lines were significantly different from those of WT at *P* < 0.01 and *P* < 0.001, respectively.

In the drought tolerance stress assay, we noticed that the WT had greater values of EL and MDA following exposure to drought conditions than did the wild type, implying that they might be subjected to more serious oxidative stress than the transgenic lines. As ROS is a major factor causing oxidative stress, therefore, we examined the accumulation of two major ROS, in particular H_2_O_2_ and $O_{2}^{-}$. Histochemical staining by DAB and NBT was used to reveal *in situ* production of H_2_O_2_ and $O_{2}^{-}$, respectively. As shown in Figure 6a, the WT accumulated dramatically more H_2_O_2_ and $O_{2}^{-}$ than both 15# and 16# under dehydration. Likewise, after drought stress the leaves of transgenic tobacco (Figure 6b) and *P. ussuriensis* (Figure 6c) were stained to a lighter extent compared with those of their respective WT lines, implying that less ROS was produced in the transgenic lines under the drought conditions. Quantitative measurements further demonstrated that H_2_O_2_ and $O_{2}^{-}$ contents in the transgenic lines of tobacco (Figure 6d,e) and *P. ussuriensis* (Figure 6f,g) were remarkably lower than those of WT. Both histochemical staining and quantitative measurement indicated that the transgenic plants were more tolerant to the oxidative stresses. The three crucial roles of antioxidant enzymes in ROS scavenging (Gill and Tuteja, 2010) prompted us to assess the enzyme activities of SOD, POD and CAT. After 7 days of withholding water, it is noticeable that the three activities of the transgenic tobacco lines were significantly higher than those in the WT (Figure 7a–c). Similarly, after drought stress the three enzyme activities of SOD, POD and CAT in the two *P. ussuriensis* transgenic lines (TG7 and TG9) were significantly higher than those of the WT (Figure 7d–f).

### Expression analysis of stress‐responsive genes in the WT and transgenic lines before and after drought

**Figure 8:**
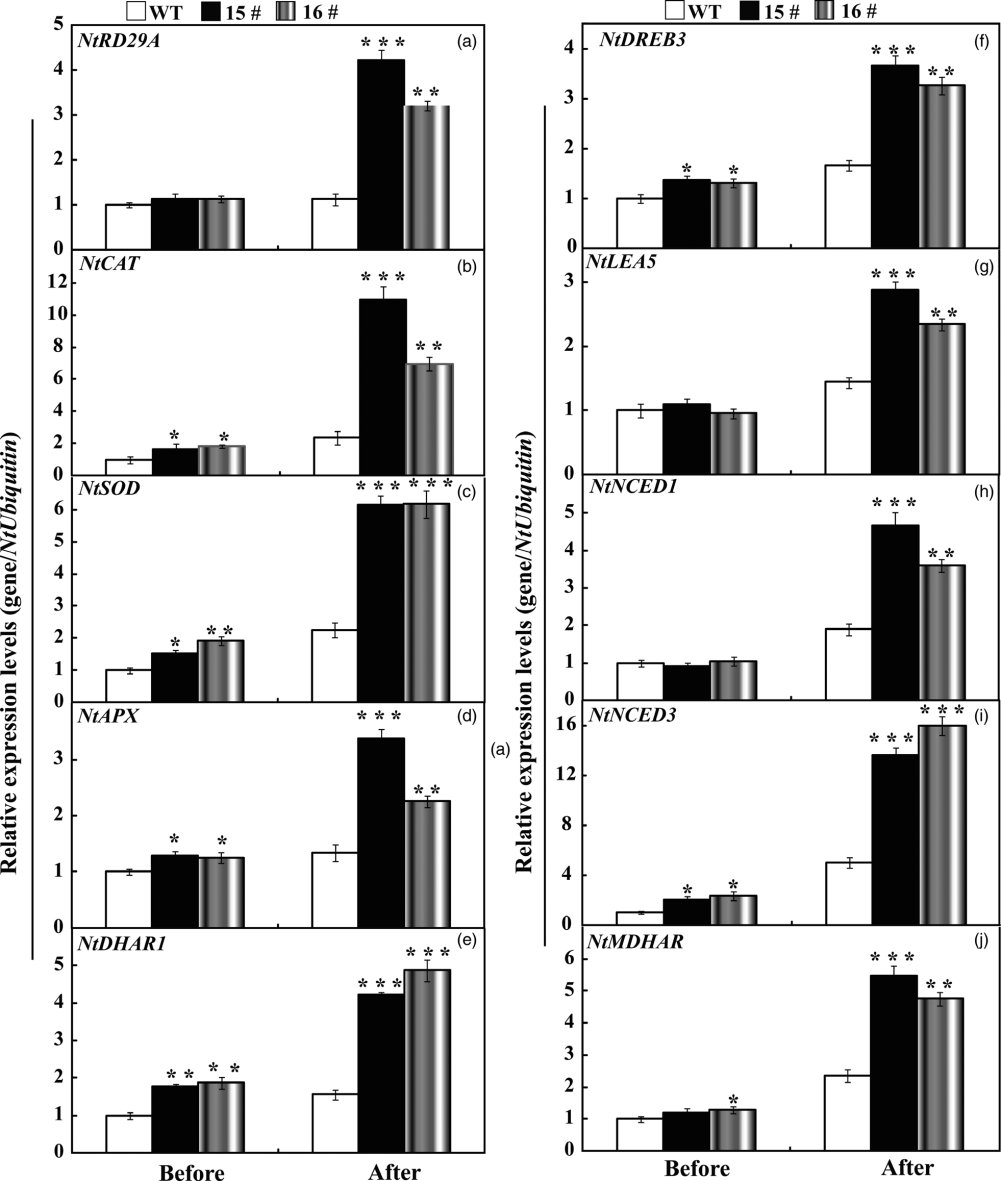
Expression profiles of the ten drought stress‐responsive genes in wild type (WT), transgenic tobacco lines (15# and 16#) before and after drought treatment. The data were analysed by Duncan's multiple range tests in the ANOVA program of SPSS (IBM SPSS 22). Asterisks show that the values are significantly different between the transgenic lines and the WT at the same time point (*P < 0.05; **P < 0.01; ***P < 0.001).

**Figure 9:**
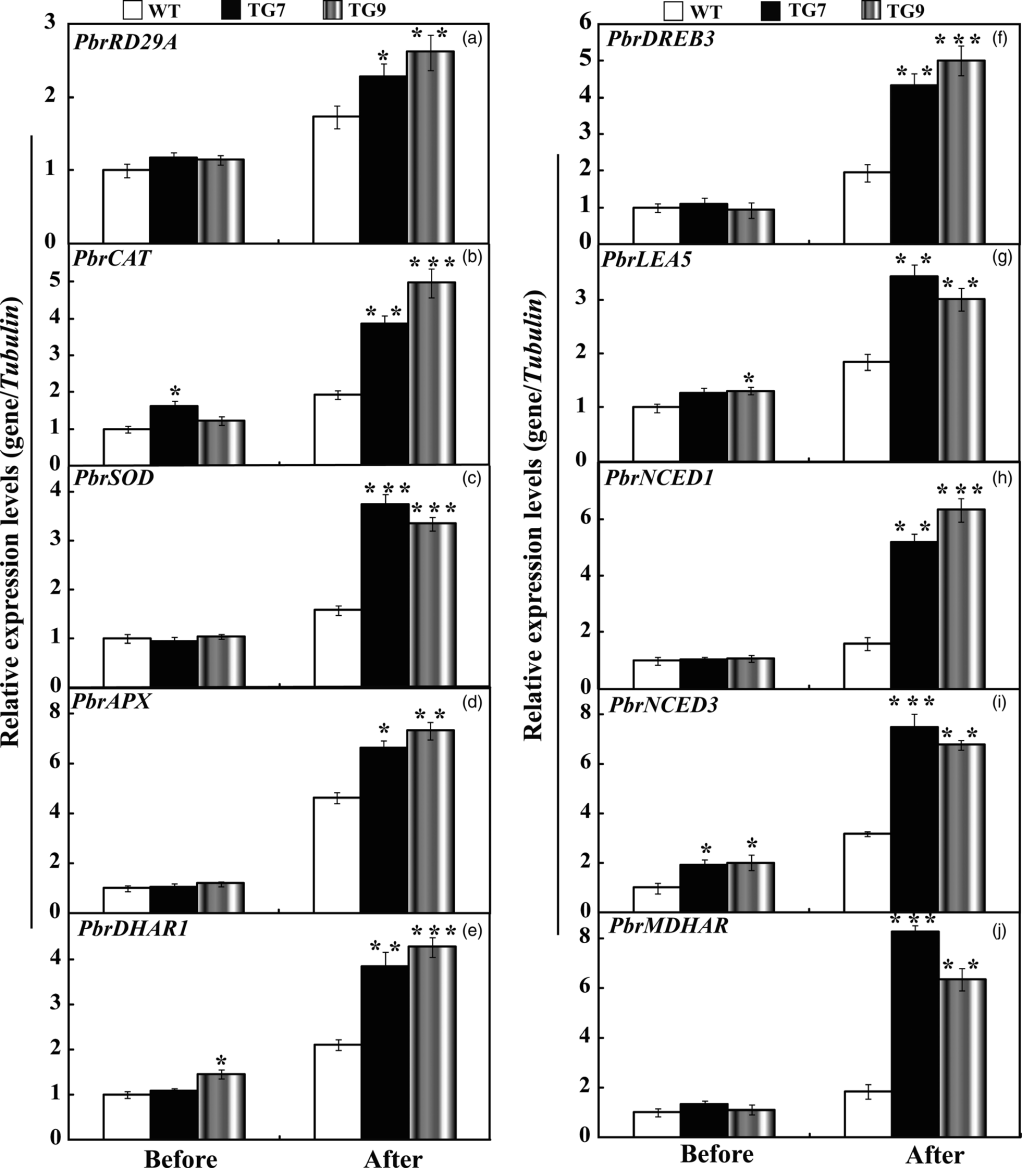
Expression profiles of the ten drought stress‐responsive genes in wild type (WT), transgenic *Pyrus ussuriensis* lines (TG7 and TG9) before and after drought treatment. The data were analysed by Duncan's multiple range tests in the ANOVA program of SPSS (IBM SPSS 22). Asterisks show that the values are significantly different between the transgenic lines and the WT at the same time point (*P < 0.05; **P < 0.01; ***P < 0.001).

To cope with unfavourable environmental constraints, plants modulate the expression of a large spectrum of stress‐responsive genes, constituting an important molecular basis for the response and adaptation of plants to stresses. To further elucidate molecular mechanism underlying the enhanced drought resistance, the transcript abundance of ten drought stress‐responsive genes was analysed by qRT‐PCR assay. Under normal conditions, mRNA levels of all ten genes in 15# and 16# were similar to those in the WT. After the drought treatment for 7 days, the transcript levels of these drought stress‐ responsive genes of transgenic lines still had more abundant expression level in comparison with the WT (Figure 8). Interestingly, exposure to drought stress caused slight up‐regulation of *NtNCED1* and *NtNCED3* in the WT, but greater induction was observed in the transgenic lines (Figure 8), which perfectly matched with the higher ABA level in transgenic lines than that of WT lines. Likewise, we also checked the transcript levels of these examined genes before and after drought stress treatments in *P. ussuriensis*. Before drought stress conditions, mRNA levels of all ten genes in TG7 and TG9 were similar to those in the WT, while the transcript levels of *PbrCAT*,* PbrSOD*,* PbrAPX* were 1.8–2.5 folds higher than that of WT (Figure 9) after drought stress. In addition, the mRNA abundance of these drought‐responsive genes in overexpression lines of *P. ussuriensis* was 1.5–5 times higher than those of WT after drought stress. It should be noticed that overexpression of *PbrWRKY53* led to higher ABA accumulation in transgenic plant than WT in *P. ussuriensis* under drought stress, which very well coincided the inducing transcript levels of *PbrNCED1* and *PbrNCED3* after withholding water for 2 weeks. This provides convincing evidence to show that the *PbrWRKY53* functions in dehydration/drought tolerance by, at least partially, enhances the transcript levels of the stress‐responsive genes, especially for *NECD1/3*.

### PbrWRKY53 directly interacts with the promoter of *PbrNCED1*

**Figure 10:**
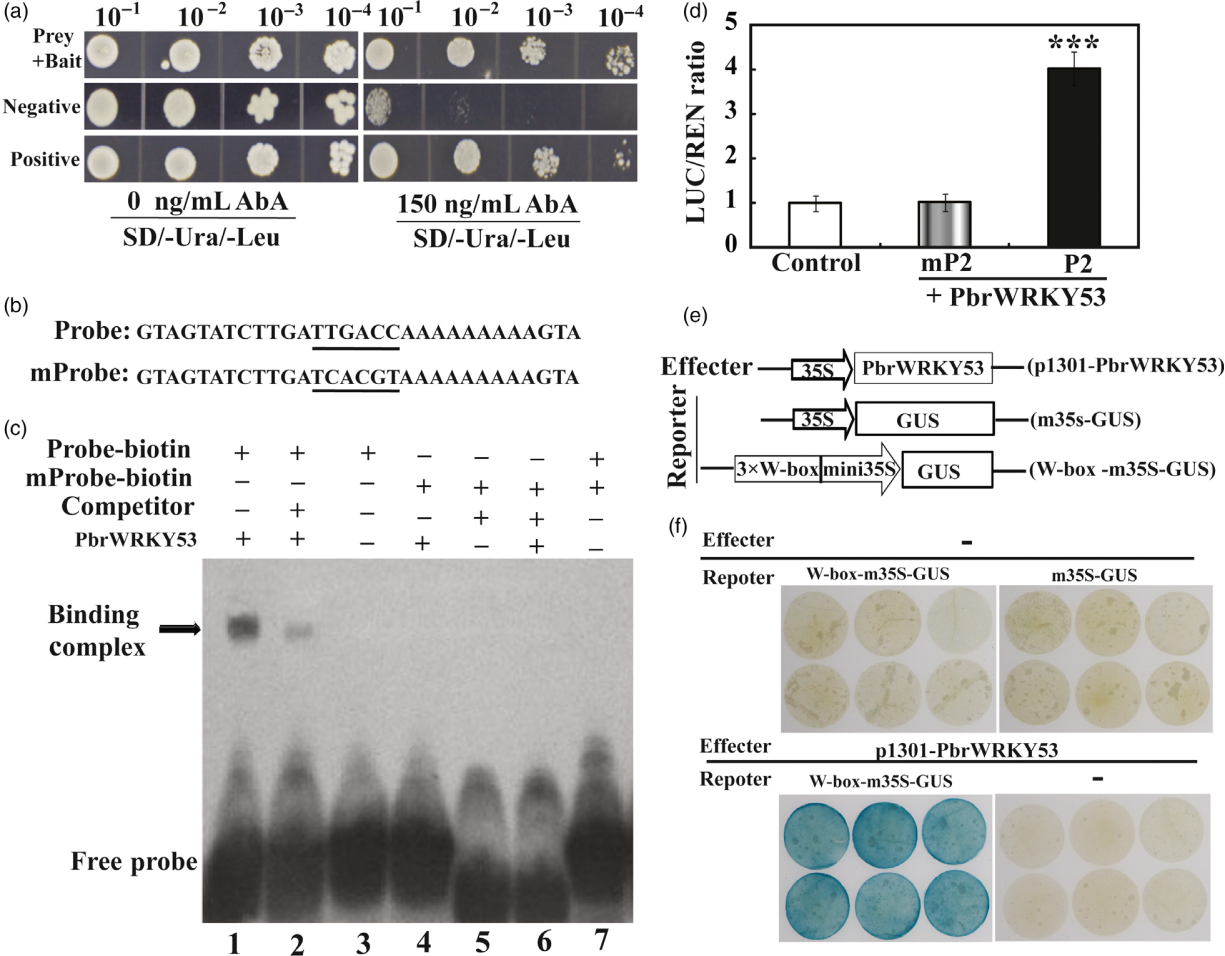
PbrWRKY53 binds to and activates the promoters of *PbrNCED1*. (a) The prey and bait vectors used for yeast one‐hybrid assay. (b) Interaction of the PbrWRKY53 protein with labelled DNA probes for the cis‐elements of the PbrNCED1 promoter in the EMSA. (c) EMSA assays using affinity‐purified fusion protein His‐PbrWRKY53 incubated with biotin‐labelled probes of *PbrNCED1* fragment containing wild type (TTGACC) or mutated W‐box element (TCACGT); the nonlabelled fragment was used as a competitor; −, absence; +, presence. (d) Transient expression assay of the promoter activity using tobacco protoplasts co‐transformed with the effector and each of the reporters containing the normal W‐box element (P2) or mP2 containing mW‐box element. (e) Schematic structures of the effector (p1301‐PbrWRKY53) and reporter vector (W‐box‐m35S‐GUS or m35S‐GUS) used for transient expression analysis. (f) GUS staining of representative leaf pieces infiltrated with only reporters (top panel) or those coinfiltrated with the effector and the reporters (W‐box‐m35S‐GUS; bottom panel). − indicates the absence of effector (top panel) or reporters (bottom panel).

Interestingly, the promoters of most of these genes contain W‐BOX (TTGACT) (Table S2). Since the expression levels of *PbrNCED1* and *PbrNCED3* were strongly induced in *PbrWRKY53*‐overexpressing lines. This promoted us to propose that these two *PbrNCEDs* might be the potential target genes that are regulated by *PbrWRKY53*. Bioinformatics analysis showed that the 2000‐bp promoter sequence of the *PbrNCED1* gene (Pbr004906.1) contains one potential W‐box element at the distal upstream region, while *PbrNCED3* had not this cis‐element in promoter region. This is designated as P1 in which the putative W‐box cis‐element is underlined. Therefore, yeast one‐hybrid (Y1H) assay was performed to examine the interaction between PbrWRKY53 and *PbrNCED1* promoter. A 198‐bp fragment containing the W‐box element was used as bait and cloned into the reporter vector, while PbrWRKY53 was used a prey. The yeast cells of positive, negative control and those co‐transformed with bait (P1)‐prey (PbrWRKY53), grew normally in screening medium. However, when 150 ng/mL AbA was added, growth of negative control was completely inhibited, while those of positive control and bait‐prey transformants were survived (Figure 10a). EMSA assay showed that formation of protein‐DNA complex was observed when His‐PbrWRKY53 was incubated with the labelled probe containing wild type W‐box element, whereas the binding was inhibited by the unlabelled competitor probe (Figure 10c). In addition, mutation of the W‐box element in the probe was completely abolished (Figure 10c). Y1H and EMSA experimental evidences together suggested that PbrWRKY53 could specifically bind to the *PbrNCED1* promoter.

To further confirm whether PbrWRKY53 could specifically bind to the *PbrNCED1* promoter. Transient expression assay was conducted using tobacco epidemical for transforming LUC reporter gene. Our results showed that the LUC/REN ratios were significantly higher in the protoplasts transformed with the effector and P2 containing reporter than those in the WT (Figure 10d). However, when W‐box element in the promoter of *PbrNCED1* was mutated, the LUC/REN ratios were resumed to the control levels (Figure 10d). To confirm the results of the Y1H assay and EMSA, transient expression analysis was conducted using PbrWRKY53 as an effector. The three repeated W‐box sequences were fused upstream of the minimal 35S GUS (m35S‐GUS) to form reporter (W‐box‐m35S‐GUS) using m35SGUS as a control reporter (Figure 10e). The leaf pieces infiltrated with only the reporters (either W‐box‐m35S‐GUS or m35S‐GUS) or the effector did not display blue colour using GUS staining, while coinfiltrated with the effector and W‐box‐m35S‐GUS were stained blue in leaf pieces. The experimental result of transient assay was consistent with the Y1H and EMSA and further confirmed the interaction between PbrWRKY53 and the W‐box element in the promoter region of the *PbrNCED1* (Figure 10f).

## Discussion

WRKY TFs comprises a large gene family in the plant genome, and form indispensable parts of regulatory networks for modulating versatile plant stress response processes (Rushton *et al*., 2010). It is worth mentioning that although increasing number of WRKYs have been comprehensively characterized their functions in the model plants (Schluttenhofer and Yuan, 2015). However, knowledge is still limited concerning the function and mode of action of plant WRKYs in woody plants especially in fruit tree *P. betulaefolia*. Therefore, functional and mechanistic characterization of WRKYs in such plants will gain better and novel insights into regulatory landscape mediated by WRKYs in stress response. In this study, excepting for isolating a WRKY TF PbrWRKY53 from *P. betulaefolia* and verified it related drought resistance of higher plants, we also further demonstrated that PbrWRKY53 is a positive regulator of *PbrNCED1* expression and AsA biosynthesis. Thus, the reported work reveals a new mechanism of PbrWRKY53 and links the function of WRKY to AsA biosynthesis.

The characteristic of WRKY genes is that each possesses one or two domains of 60 amino acids with highly conserved WRKYGQK motif at N terminus, and at C terminal contains Cys2His2 or Cys2HisCys zinc‐finger motif (Eulgem and Somssich, 2007; Rushton *et al*., 2010). WRKYs are categorized into three major groups (I, II and III), in which group II is further divided into five subgroups, IIa, IIb, IIc, IId and IIe, based on number of WRKY domains and structure of zinc finger motif (Rushton *et al*., 2010). According to this unique signature, PbrWRKY53 is classified into Group III category. The Group III WRKYs, such as *Arabidopsis thaliana* ABO3/WRKY63, and WRKY57 have been previously shown to play positive roles in regulating abiotic stress response (Jiang *et al*., 2012; Ren *et al*., 2010). Conversely, in *A. thaliana*,* wrky46wrky54wrky70* triple mutant showed more tolerant to drought stress by modulating brassinosteroids (BR)‐regulated plant growth and promoted expression levels of drought‐responsive genes, which indicated AtWRKY46, AtWRKY54 and AtWRKY70 were negative regulator of drought tolerance (Chen *et al*., 2017). In this study, overexpression of *PbrWRKY53* conferred enhanced tolerance to dehydration and drought stresses, indicating that PbrWRKY53 is acted as positive regulator of dehydration and drought tolerance. Our findings and early studies showed that WRKY genes in class III play a key role in abiotic stress, and thus hold great potential in improving plant stress tolerance and breeding improved seeds.

As dehydration stress resulted in a stronger induction of *PbrWRKY53* transcript level than salt and cold, we made efforts to elucidate its function in dehydration/drought tolerance by generating transgenic plants transformed with either tobacco or *P. ussuriensis*, respectively. In this study, the transgenic lines overexpressing *PbrWRKY53* displayed better phenotypic morphology, concomitant with less water loss, lower EL, lower levels of MDA and higher chlorophyll content than WT under either short‐ or long‐term water stress (dehydration and drought), suggesting that overexpression of *PbrWRKY53* conspicuously conferred tolerance to drought stress. In line with previous studies, overexpression of *WRKY* genes in either model or non model plants have been shown to render tolerance to abiotic stresses (Dai *et al*., 2018; Dang *et al*., 2013; Gong *et al*., 2015; Hu *et al*., 2013; Jiang *et al*., 2012; Ren *et al*., 2010; Zhou *et al*., 2008), demonstrating that WRKY genes might hold great potential for stress tolerance.

Because it is well known that in biological systems ROS accumulation is related to physiological perturbation and ROS accumulation depends greatly on the balance between production and concurrent scavenging (Buchanan and Balmer, 2005; Miller *et al*., 2010; Pitzschke *et al*., 2009; Suzuki *et al*., 2012). In order to elucidate the physiological mechanism underlying the enhanced drought tolerance remained, which stimulated us to conduct more work on comparing their ROS levels. Both histochemical staining by DAB and NBT together demonstrated that lower levels of two major types of ROS, H_2_O_2_ and $O_{2}^{-}$, were observed in the transgenic lines than in the WT when they were treated with dehydration or drought. Plant cells possess a complex antioxidant defense system for ROS detoxification, which is achieved by either ROS‐scavenging enzymes, such as SOD, CAT, POD or non‐enzymatic antioxidants like AsA and APX (Miller *et al*., 2010). It was observed that activities of these enzymes such as AsA and APX, and higher activities of SOD, POD and CAT, were significantly higher in the transgenic plants either tobacco or *P. ussuriensis* than those of their respective WT lines. This indicates that overexpression of the *PbrWRKY53* gene has more robust detoxifying system to eliminate ROS produced during stress.

To gain a deeper understanding of the function of *PbrWRKY53* in stress tolerance at molecular level, transcript levels of ten stress‐responsive genes were monitored before and after drought treatment, including *NtRD29A/PbrRD29A*,* NtCAT/PbrCAT*,* NtSOD/PbrSOD*,* NtAPX/PbrAPX*,* NtDHAR1/PbrDHAR1*,* NtDREB3/PbrDREB3*,* NtLEA5/PbrLEA5*,* NtNCED1/PbrNECD1*,* NtNCED3/PbrNECD3* and *NtMDHAR/PbrMDHAR* which or whose homologues in other plants have been shown to be involved in abiotic stress response. Our results showed transcript levels of the genes encoding ROS‐scavenging enzymes (*NtCAT/PbrCAT*,* NtSOD/PbrSOD*) were up‐regulated in the transgenic lines before and after drought, consistent with the greater activity of these antioxidant enzymes. This may presumably explain the activation of the antioxidant enzymes and the lower ROS levels in the transgenic lines. On the other hand, three AsA‐GSH cycle genes (*DHAR1*,* MDHAR* and *APX*) involved in AsA synthesis were also induced to a higher level in the transgenic lines in tobacco (15# and 16#) and *P. ussuriensis* (TG7 and TG9) relative to their respective WT lines (Figures 8 and 9). Circumstantial evidence has demonstrated ROS can be transformed into H_2_O_2_, and the AsA‐GSH recycling pathway, can scavenge by converting it into H_2_O using AsA (Mittova *et al*., 2000). AsA is an important component of the plant antioxidant system that plays critical roles in regulating ROS levels and dealing with abiotic stress (Chen and Gallie, 2005; Hemavathi *et al*., 2010). Stronger induction of these genes (*DHAR1*,* MDHAR* and *APX*) suggests that the transgenic lines may produce higher levels of AsA (Figures 4 and 5), resulting in the reduced damage to the plants, which may presumably explain the lower levels of ROS in the transgenic lines (Figure 6). The transgenic lines displayed lower water loss rates than the WT under dehydration, suggesting that the former may close their stomata more sufficiently. On the other hand, higher survival rate was detected in the transgenic lines than in the WT after a period of drought treatment using potted plants. It implies that the transgenic plants might preserve the water under stress in a better way. This assumption was supported, at least in part, by the assessment of expression profiles of the late embryogenesis abundant (LEA) genes, *NtLEA5/PbrLEA5. NtLEA5/PbrLEA5* encodes LEA protein that is assumed to play critical roles in combating cellular dehydration (Hundertmark and Hincha, 2008). In addition to the functional genes, it is noted that the *NtRD29A/PbrRD29A*,* NtDREB3/PbrEREB3*,* NtNCED1/PbrNCED1* and *NtNCED3/PbrNCED3* were also induced to higher levels in the transgenic lines before and after drought stress condition (Figure 9).

**Figure 11:**
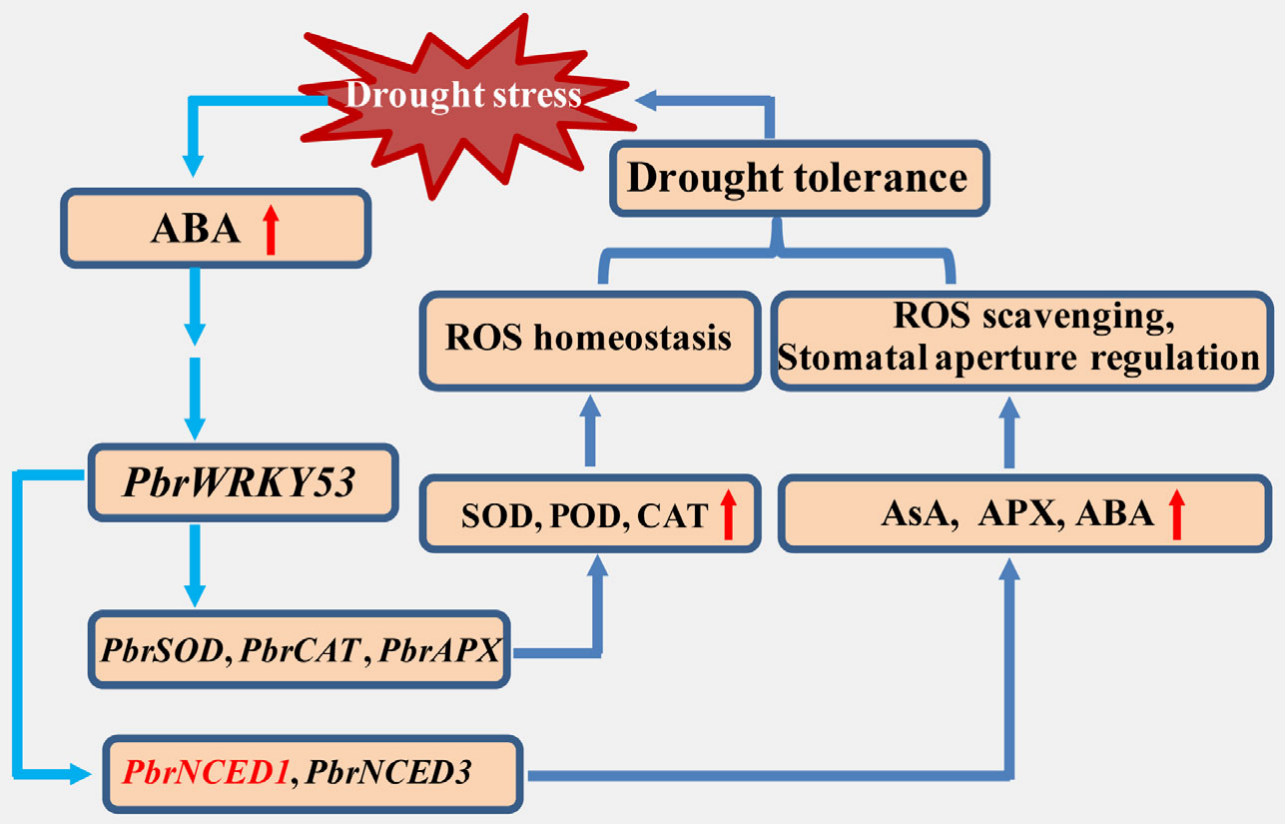
A proposed model of action for demonstrating regulatory function of *PbrWRKY53* in response to drought stress. The ABA signalling pathway is triggered by drought stress, leading to enhancing the expression levels of *PbrWRKY53*, which in turn regulates *PbrNCED1* through binding to W‐box element in its promoter. Up‐regulation of the PbrWRKY53‐PbrNCED1 module promotes AsA synthesis and ROS scavenging, allowing the drought‐associated damage to be alleviated by maintaining ROS homeostasis and adjustment of stomatal aperture.

Induction of their mRNA to higher levels raised the possibility of interaction between them and *PbrWRKY53* in order to orchestrate well‐defined stress tolerance machinery that functions in protection of the plants against adverse environment. Interestingly, two genes (*NtNCED1/PbrNCED1* and *NtNCED3/PbrNCED3*) involved in ABA synthesis in higher plants displayed significantly higher mRNA abundance compared with the other stress‐related genes. It has been reported that *NCED1* is predominantly responsible for ABA accumulation under drought, while *NCED3* knockout mutant exhibit drought‐sensitive phenotype with reduced ABA level (Iuchi *et al*., 2001; Xian *et al*., 2014). In this work, a W‐Box recognizing element was revealed in the promoter of *PbrNCED1* and Y1H, transient expression assays and EMSA together provided solid evidences further supporting a direct and specific interaction between PbrWRKY53 and the *PbrNCED1* promoter. These data enable us to surmise that *PbrNCED1* is a target gene of PbrWRKY53, suggesting that *PbrNCED1* can be assigned to the WRKY regulon. This is partially supported by an earlier work in which overexpression of *NCED1* could improve vitamin C level and tolerance to drought in transgenic tobacco (Bao *et al*., 2016). Our data showed that *PbrWRKY53* acts as a positive regulator of drought tolerance, which may be partly ascribed to its role in modulating vitamin C level by regulating *NCED1* expression. Here, we propose a model of action for the regulatory function of *PbrWRKY53* in response to drought stress based on our findings (Figure 11). Drought stress triggers the ABA signalling pathway, leading to enhancing the expression of *PbrWRKY53*, which in turn regulates *PbrNCED1* through binding to W‐box elements in its promoter. Up‐regulation of the PbrWRKY53‐PbrNCED1 module promotes AsA synthesis and ROS scavenging, allowing the drought‐associated damage to be alleviated by maintaining ROS homeostasis and adjustment of stomatal aperture. This study provides a novel insight into the molecular mechanisms underlying AsA synthesis and ROS scavenging in response to drought stress.

In conclusion, we identified a novel drought‐responsive WRKY TF, *PbrWRKY53*, isolated from *P. betulaefolia*, which acts as a positive regulator of drought tolerance. The *PbrWRKY53‐*overexpression plants accumulated more AsA levels, which in turn activated its downstream *PbrNCED1*. In addition, bioinformatics prediction revealed the presence of one W‐box element on the promoter of *PbrNCED1* gene, which were shown to be interacted by PbrWRKY53, indicating that *PbrNCED1* might serve as a direct target gene of PbrWRKY53. Y1H, transient expression assays and EMSA provided evidence further supporting a direct and specific interaction between PbrWRKY53 and the *PbrNCED1* promoter. Taken together, these results demonstrated that *PbrWRKY53* functioned in mediating drought tolerance by elevation of AsA levels via regulating *PbrNCED1* gene, which may explain the higher levels of *NCED1* and AsA level in the transgenic plants of tobacco and *P. ussuriensis*. Establishment of the WRKY‐NCED network provides new valuable knowledge of the function and underlying molecular mechanism of WRKY and expands our understanding of the complex drought signalling network. However, In the future, extra work is needed to decipher other components related to *PbrWRKY53* so as to gain a clear‐cut the molecular mechanisms underlying *PbrWRKY53* function in drought tolerance.

## Materials and methods

### Plant materials and stress treatments

Healthy shoots from 45‐day‐old *P. betulaefolia* plants grown in the greenhouse of Nanjing Agricultural University were used for gene cloning and expression analysis of *PbrWRKY53* under various stress treatments. The shoots were washed and cultured for 1 day in a growth chamber to minimize the mechanical stress on the tissues, followed by exposure to corresponding stress treatments, which were carried out as follows. For dehydration treatment, the shoots were placed on dry filter papers for 0, 0.5, 1, 3 and 6 h at ambient environment. For salt stress, the seedlings were treated with 200 mm NaCl solution for 0, 1, 3, 6, 12 and 24 h. In addition, for cold stress, the shoots were moved to a growth chamber set at 4 °C for 0, 1, 5, 12, 24 and 48 h. For each treatment, at least 15 seedlings were used and the leaves were sampled from three randomly collected seedlings at designated time point and frozen immediately in liquid nitrogen and stored at −80 °C until use.

### Isolation and bioinformatics analysis of *PbrWRKY53*

In an earlier study, based upon a drought‐induced WRKY TF (Pbr018725.1) showing high sequence homology to WRKY53 was found to be up‐regulated in the transcriptome (Li *et al*., 2016). A pair of gene‐specific primers (GSP1, Table S1) was designed based on the sequence so as to amplify *PbrWRKY53* in *P. betulaefolia*. For this purpose, total RNA was isolated from dehydration‐treated leaves using TRIZOL reagent (Takara Bio Group, Dalian, China) and treated with RNase‐free *DNase* I (Takara Bio Group) to remove DNA contamination. Approximately 1 μg of total RNA was reversely transcribed into cDNA using RevertAid First Strand cDNA Synthesis Kit (TOYOBO, Osaka, Japan). PCR mixture, in a total volume of 50 μL, contained 200 ng of cDNA, 1× reaction buffer, 2.5 mm MgCl_2_, 0.25 mm dNTP, 2.5 units of TransStart FastPfu DNA polymerase, and 0.5 μm of each primer. The PCR product was purified, cloned into pMD18‐T vector (Takara Bio Group) and sequenced (Sunny, Shanghai, China). Homology search was performed in NCBI website, while sequence alignments were conducted using Clustal W and Genedoc software. A phylogenetic tree was constructed using and MAGA 4.0 software (www.megasoftware.net/mega4/). Theoretical isoelectric point (*p*I) and molecular weight were predicted in an internet server, ExPASy (Expert Protein Analysis System, http://www.expasy.org/tools).

### Subcellular localization of PbrWRKY53

The complete ORF sequences of PbrWRKY53 without a stop codon was amplified by RT‐PCR using GSP2 (Table S1) containing single restriction sites of *Nco* I. The purified PCR products were digested with *Nco* I and then fused to the pCAMBIA1302 vector to get a fusion protein PbrWRKY53::GFP under the control of the CaMV 35S promoter. For construction of 35S::nls::mKate::RFP vector, the nuclear signal peptide (MDPKKKRKV) was fused to the far‐RFP mKate (Shcherbo *et al*., 2007) of pBWA(V)HS vector driven by CaMV 35S promoter. Both PbrWRKY53::GFP and 35S::nls::mKate::RFP were cotransformed into rice protoplasts isolated from etiolated shoots via polyethylene glycol treatment as previously described by Zhang *et al*. (2011). The construct 35S::GFP was used as a control. The fluorescence signal was observed with a confocal microscope (Leica) after transformation for 12–16 h. GFP signal was excited using a wavelength of 488 nm. The RFP emission signal was collected between 587 and 610 nm.

### Generation of transgenic plants and *P. ussuriensis* silenced plants

The full‐length cDNA of *PbrWRKY53* gene was PCR‐amplified with GSP3 (Table S1) and inserted into binary vector pCMABIA1301 linearized with *Nco* I *or BstE* II, under the control of the *CaMV 35S* promoter. Virus‐induced gene silencing (VIGS)‐mediated suppression of PbrWRKY53 was performed according to previous methods (Jiang *et al*., 2014; Wang *et al*., 2016). For the construction of pTRV2‐PbrWRKY53, a 487 bp fragment of the *PbrWRKY53* ORF (52–531 bp) was inserted into *Xba* I and *Sac* I sites of tobacco rattle virus‐based vector 2 (TRV2) to generate the pTRV2‐PbrWRKY53 construct. Empty pTRV2 vector was used as a control. All the recombinant plasmids were introduced into *Agrobacterium tumefaciens strain* GV3101 by heat shock. *Agrobacterium*‐mediated transformation of tobacco (*Nicotiana tabacum*) and *P. ussuriensis* was performed as described (Huang *et al*., 2010; Li *et al*., 2017 and Yang *et al*., 2017). The overexpression plants with hygromycin‐resistant were verified by amplifying the sequences of hygromycin genes in tobacco or *P. ussuriensis*. Overexpression of *PbrWRKY53* was assayed by semi‐quantitative PCR in positive transgenic tobacco plants (Figure S1). T_2_ seeds of overexpressing lines were used for the subsequent stress tolerance assay. These overexpression lines of *P. ussuriensis* were used for Western blotting assays with an anti‐Flag antibody (Figure S2F). The reference genes, *Tubulin* and *Ubiquitin*, were used as internal controls for *P. ussuriensis* and tobacco, respectively.

### Assessment of drought tolerance in transgenic lines

The WT and transgenic lines were subjected to dehydration and drought in order to examine their stress tolerance capacity. For dehydration treatment, leaves excised from 30‐day‐old seedlings of tobacco transgenic lines were put on clean filter papers and allowed to dry for up to 60 min. Fresh weights of the leaves were measured at the designated times, and the water loss rate was calculated by comparison with the initial weight. The leaves were sampled at the completion of dehydration and used for the examination of stomatal apertures and EL. For the drought tolerance assay, two sets of experiments were designed. First, 18‐day‐old soil‐grown tobacco transgenic plants were deprived of water for 15 days and then returned to regular irrigation for 3 days. Survival rates were then scored. In the second set of experiment, 65‐day‐old tobacco transgenic plants were subjected to drought by ceasing irrigation for 7 days; at the end of treatment, the leaves were collected for measurement of EL and MDA content. However, leaves sampled at the onset of and 7 days after drought were frozen immediately in liquid nitrogen and stored at −80 °C until use for analysis of enzyme activity and gene expression. Two *P. ussuriensis* overexpression lines (TG7 and TG9) and WT plants were subjected to drought by ceasing irrigation for 2 weeks. EL, MDA and antioxidant enzyme activities in the leaves at the last time point were examined as described above.

The dehydration treatment was repeated at least three times, while drought and osmotic stress treatment were repeated twice. Three replicates were used for each line under the stress treatment.

### Isolation of *PbrNCED1* promoter and PbrWRKY53 and Y1H assay

Promoter sequence of *PbrNCED1* was obtained by genomic PCR with primer GSP4 (Table S1) using *P. betulaefolia* genomic DNA as template. Potential *cis*‐acting elements related to drought response and transcription start site were predicted on PLACE (http://www.dna.affrc.go.jp/PLACE/) and BDGP (http://www.fruitfly.org/seq_tools/promoter.html), respectively. To this end, the full‐length ORF of *PbrWRKY53* without a stop codon was RT‐PCR amplified using GSP5 (Table S1) and integrated into the *EcoR* I and *Xho* I sites of pGADT7 to generate the effector vector pGADT7‐PbrWRKY53. Meanwhile, based on the distribution of the W‐box sequence, one fragment (P1, −727 to −721) was amplified using primers (GSP6, Table S1) containing *Sma* I and *Xho* I restriction site and subcloned to the pAbAi to generate a reporter vector pAbAi‐PbrNCED1. Yeast one‐hybrid assay was used to examine the interaction of PbrWRKY53 and the *PbrNCED1* promoter according to the manual provided by Matchmaker Gold Y1H Library Screening System (Clontech, Dalian, China). The yeast cells co‐transformed with the prey and either of the bait was cultured for 3 days on SD/‐Ura/‐Leu medium added with or without 150 ng/mL Aureobasidin A (AbA).

### EMSA

The CDS of *PbrWRKY53* was amplified and inserted into the pCzn1‐His vector to generate the recombinant His‐6‐PbrWRKY53 protein. The resulting construct was expressed in *Escherichia coli* strain BL21 (DE3) cells, and the recombinant protein was expressed and purified using Ni‐IDA resin according to the manufacturer's instructions. EMSAs were performed using the Light Shift Chemiluminescent EMSA Kit (Pierce, IL) according to the manufacturer's protocol and as described (Wu *et al*., 2016). A 30 bp biotin‐labelled DNA probes containing either WT (P1) or mutated W‐box element (mP1), and unlabelled competitor DNA were synthesized (Shanghai Sangon Biotechnology, Shanghai, China) based on the *PbrNCED1* promoter sequence and labelled using the Biotin 3′ End DNA Labelling Kit (Thermo Fisher Scientific, Waltham, MA, USA). The binding reaction was performed for 20 min at room temperature in a 20 μL reaction buffer. The DNA–protein complexes were separated on 6.5% polyacrylamide gel and electrophoretically transferred to a nylon membrane (GE Healthcare, Danderyd, Sweden), and detected following the manufacturer's instructions. This experiment was conducted three times.

### Transient expression assay

The coding region of *PbrWRKY53* was amplified using primers (GSP7, Table S1) containing with *Sma* I and *Xho* I restriction site and inserted into pGreenII 62‐SK linearized with the same enzymes to yield an effector plasmid. A 198‐bp *PbrNCED1* promoter fragment was PCR amplified with specific primers [GSP8 (P2), Table S1] containing either *Pst* I or *BamH* I restriction sites and ligated into the reporter vector, pGreen II 0800‐LUC (Hellens *et al*., 2005). The W‐box element in P2 was mutated by PCR (mP2, Table S1). The effector and reporter constructs were transformed into *A. tumefaciens* GV3101 cells. Assays for transient expression in protoplasts were performed as described previously (Agarwal *et al*.,^2006^; Yoo *et al*., 2007) with minor modifications. The transformed protoplasts were incubated overnight under dark at 25 °C for 18 h before analysis of firefly luciferase (LUC) and *Renilla* Luciferase (REN) via the Dual‐Luciferase^®^ Reporter Assay System (Promega, Madison, WI, USA) with an Infinite200 Pro reader (Tecan, Männedorf, Switzerland). The promoter activity was expressed as the ratio of *LUC*/*REN*, and the *LUC*/*REN* ratio of which was set as 1. The transient promoter expression assay was carried out to confirm the interaction between PbrWRKY53 and the W‐box element using a method modified from that of (Li *et al*., 2010).The minimal‐100 CaMV 35S (m35S) was amplified by RT‐PCR from the pBI221 vector using primers consisting *Hind* III or *BamH* I restriction sites (GSP9, Table S1), then connected with the upstream of the GUS reporter gene of pCAMBIA1391 to produce the m35S‐GUS recombinant construct. Three repetitions of W‐box element, containing *Pst* I and *EcoR* V two restriction sites and were inserted upstream of the m35S sequence of pBI221 to generate W‐box‐m35S‐GUS‐pBI221, which was digested by *Pst* I and *Xba* I and subcloned into pCAMBIA1301 to get a recombinant construct of W‐box‐m35S‐GUS‐pCAMBIA1301. Then, the constructs were inserted into the pCAMBIA1391 vector at the *Hind* III and *BamH* I sites to produce the reporter constructs (W‐box‐m35S‐GUS), which were transformed into *A*. *tumefaciens* (GV3101). The overexpression‐PbrWRKY53 construct was used as the effector. *A. tumefaciens*‐mediated transformation of tobacco leaf pieces was conducted as described (Huang *et al*., 2010) with the exception of using the *A. tumefaciens* cells harboring the effector and the reporter at a 1:1 ratio, whereas only the infection with m35S‐GUS or the effector was acts as the control. After incubated in the dark for 2 days at 25 °C, the leaf pieces were used for measuring GUS activity by histochemical method according to (Jefferson *et al*., 1987). The transient expression assay was performed with three times and same results were observed at each time.

### Analysis of EL, MDA, antioxidant enzyme activity and metabolite levels

Electrolyte leakage was measured as described (Dahro *et al*., 2016). MDA content, and CAT, POD and SOD activities, expressed as units/mg protein, were measured using analytical kits (Nanjing Jiancheng Bioengineering Institute, Nanjing, China). Proline content was assessed as described (Zhao *et al*., 2009) with slight modification. Extraction of ABA in the leaf samples collected was performed as essentially described in a previous work (Liu *et al*., 2012). AsA and DHA contents were measured essentially as described (Li *et al*., 2009).

### Statistical analysis

Each stress treatment was repeated at least three times with consistent results. Data are presented as means ± SE of at least three independent replicates from one representative experiment. The data were analysed by Duncan's multiple range tests in the ANOVA program of SPSS (IBM SPSS 22, Chicago, IL, USA), taking *P* < 0.05, *P* < 0.01, *P* < 0.001 as significantly different.

## Conflict of interest

The authors declare no conflict of interest.

## Supporting information


**Figure S1** Generation and molecular identification of transgenic tobacco plants overexpressing PbrWRKY53.
**Figure S2** Generation and molecular identification of transgenic *Pyrus ussuriensis* plants overexpressing PbrWRKY53.
**Figure S3** Silencing of PbrWRKY53 by virus‐induced gene silencing (VIGS) leads to impaired drought tolerance in *Pyrus ussuriensis*.
**Table S1** Primer sequences used for cloning, subcellular localization, vector construction, transgenic confirmation and expression analysis.
**Table S2** Analysis of stress‐responsive genes promoter W‐BOX element.
