# Supplementary material for: A WRKY transcription factor PbrWRKY53 from Pyrus betulaefolia is involved in drought tolerance and AsA accumulation
Source: Plant Biotechnol J. 2019 Mar 19;17(9):1770–87. doi: 10.1111/pbi.13099 (PMC6686137; doi:10.1111/pbi.13099)
Supplement: Supplementary file 1 — Figure S1 Generation and molecular identification of transgenic tobacco plants overexpressing PbrWRKY53. Figure S2 Generation and molecular identification of transgenic Pyrus ussuriensis plants overexpressing PbrWRKY53. Figure S3 Silencing of PbrWRKY53 by virus‐induced gene silencing (VIGS) leads to impaired drought tolerance in Pyrus ussuriensis. Table S1 Primer sequences used for cloning, subcellular localization, vector construction, transgenic confirmation and expression analysis. Table S2 Analysis of stress‐responsive genes promoter W‐BOX element. [file PBI-17-1770-s001.docx]

**Supporting information**


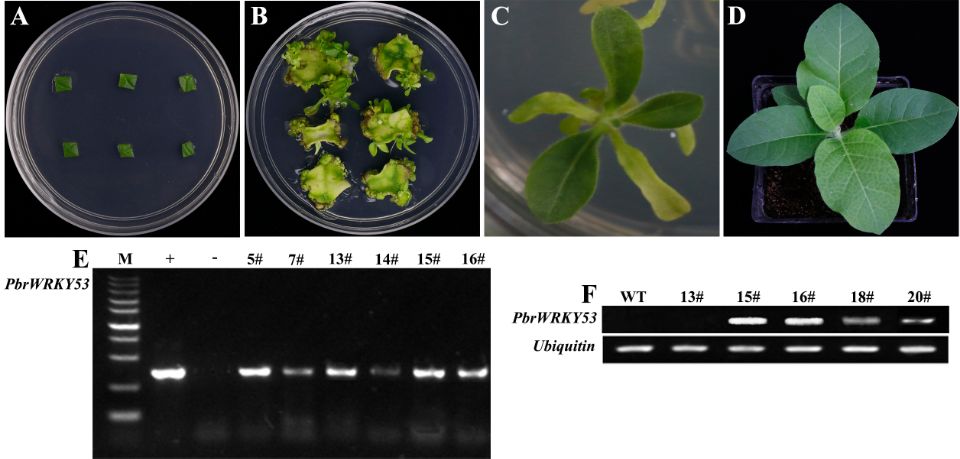


Figure S1. Generation and molecular identification of transgenic tobacco plants overexpressing *PbrWRKY53*. (A-D) Genetic transformation process of the tobacco leaf discs via *Agrobacterium*-mediated transformation of the overexpression vector. (E) PCR identification of the regenerated plants using specific primers of *PbrWRKY53*. M, DNA marker (M). +, positive control (plasmid). -, negative control (wild type). The numbers indicated different transgenic lines. (F) Semi-quantitative RT-PCR analysis of the mRNA levels of *PbrWRKY53* in the five transgenic lines and wild type (WT).


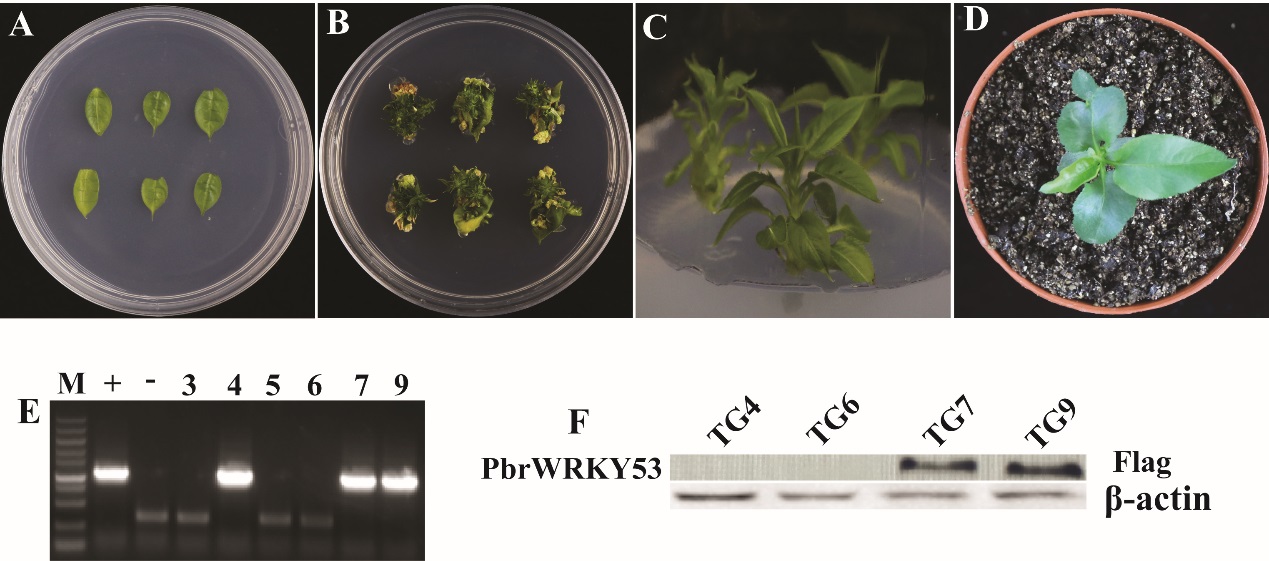


Figure S2. Generation and molecular identification of transgenic *Pyrus ussuriensis* plants overexpressing *PbrWRKY53*. (A-D) *Agrobacterium*-mediated transformation of process of the pear leaf discs and plant regeneration. (E) PCR identification of the regenerated buds using CaMV35S- PbrWRKY53 primers. M: DNA Marker; +: plasmid; -: WT (wild type); the numbers indicated different transgenic lines. (F) Western blotting analysis of *PbrWRKY53* proteins in the wild-type and transgenic *Pyrus ussuriensis* plants. β-*actin* served as a protein loading control for transgenic pear plants.


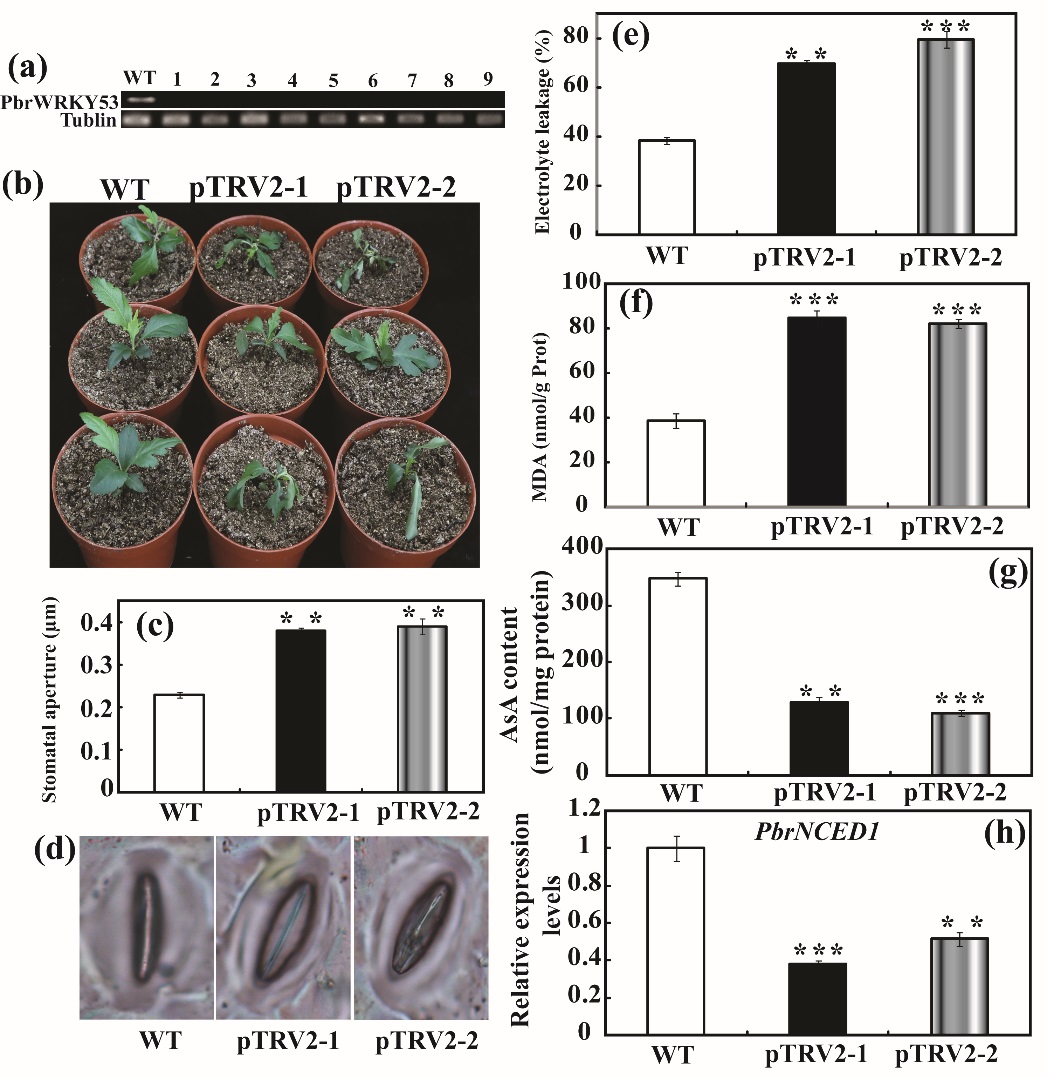


Figure S3. Silencing of *PbrWRKY53* by virus-induced gene silencing (VIGS) leads to impaired drought tolerance in *Pyrus ussuriensis*. (a) Genomic PCR for identification of the plants derived from the agro-infiltration. (b) Phenotypes of *PbrWRKY53*‐silenced plants (b) after drought. (c–d) Stomatal aperture size (c) and representative images (d) of pTRV (WT) plants and pTRV2-PbrWRKY53 silencing plants after drought. (e-g) Electrolyte leakage (e), MDA levels (f) and AsA content (g) of the pTRV2 (WT) plants and pTRV2-PbrWRKY53 silencing plants after drought. (h) Expression profile of *PbrNCED1* gene in *Pyrus ussuriensis* silenced plants under drought conditions. Asterisks show that the values are significantly different between the transgenic lines and the WT at the same time point (**P <0.01; ***P <0.001).

Table S1 Primer sequences used for cloning, subcellular localization, vector construction, transgenic confirmation and expression analysis

| **Genes** | **Primers** | **Sequences (**5ʹ-3ʹ**)** | |
| --- | --- | --- | --- |
|  |  | **Forward** | **Reverse** |
| *PbrWRKY53* | GSP1 | ATGGAGAACTGCAATATACATTGGGAG | TTAGGAAAAGAATCCTGGACCGTC |
| *PbrWRKY53*  *PbrWRKY53*  *PbrNCED1*  *PbrWRKY53*  *PbrNCED1*  *PbrWRKY53*  *PbrNCED1*  Transient expression assay | GSP2  GSP3  GSP4  GSP5  GSP6  GSP7  GSP8  GSP9 | CCATGGATGGAGAACTGCAATATACATTGGGAG (*Nco*I site is underlined)  CCATGGCTCAGCTTACCCTTCTCACAAATCTG (*Nco*I site is underlined)  CATTTCAGCTTCAGAATTTTGAGACGCAACGG  GAATTCATGGAGAACTGCAATATACATTGGGAG (*EcoR* I site is underlined)  CCCGGGGCACTTCTTATGACACATGCCAGTTACC (*Sma* I site is underlined)  CCCGGGATGGAGAACTGCAATATACATTGGGAG (*Sma* I site is underlined)  CTGCAGGCACTTCTTATGACACATGCCAGTTACC (*Pst* I site is underlined)  CAGGATCCGATATCTCCACTGACGTAAGGG (*Bam*HI site is underlined) | CCATGGGGAAAAGAATCCTGGACCGTC (*Nco*I site is underlined)  GGTGACCGGAAAAGAATCCTGGACCGTC (*BstE* II site is underlined)  ATCAGTGCTGTGCAAAACAGACGAGTGAAAGTG  CTCGAGGGAAAAGAATCCTGGACCGTC (*Xho* I site is underlined)  CTCGAGGGTCAGACTATTGAAAGTTCGATTAC (*Xho* I site is underlined)  CTCGAGGGAAAAGAATCCTGGACCGTC (*Xho* I site is underlined)  GGATCCGGTCAGACTATTGAAAGTTCGATTAC (*BamH* I site is underlined)  GGAAGCTTCGTGTTCTCTCCAAATGAAATG  (*Hin*dIII site is underlined) |
| ***Tublin*** |  | TGGGCTTTGCTCCTCTTAC | CCTTCGTGCTCATCTTACC |
| ***Ubiquitin*** |  | AGCTACATGACGCCATTTCC | CCCTGTAAAGCAGCACCTTC |

Table S2 Analysis of stress-responsive genes promoter W-BOX element.

| Gene | GeneBank ID | related element | Quantity |
| --- | --- | --- | --- |
| RD29A | Pbr004104.1 | W-BOX(TTGACT) | 2 |
| CAT | Pbr007743.1 | W-BOX(TTGACT) | 1 |
| SOD | Pbr034143.1 | W-BOX(TTGACT) | 0 |
| DHAR1 | Pbr032314.1 | W-BOX(TTGACT) | 1 |
| DREB3 | Pbr008796.1 | W-BOX(TTGACT) | 1 |
| LEA5 | Pbr014877.1 | W-BOX(TTGACT) | 2 |
| APX | Pbr008291.1 | W-BOX(TTGACT) | 3 |
| NCED1 | Pbr004906.1 | W-BOX(TTGACT) | 1 |
| NCED3 | Pbr025271.1 | W-BOX(TTGACT) | 0 |
| MDHAR | Pbr017802.1 | W-BOX(TTGACT) | 2 |
